# Statistical Genomics Analysis of Simple Sequence Repeats from the *Paphiopedilum Malipoense* Transcriptome Reveals Control Knob Motifs Modulating Gene Expression

**DOI:** 10.1002/advs.202304848

**Published:** 2024-04-22

**Authors:** Yingyi Liang, Jing Hao, Jieyu Wang, Guoqiang Zhang, Yingjuan Su, Zhong‐Jian Liu, Ting Wang

**Affiliations:** ^1^ College of Life Sciences South China Agricultural University Guangzhou 510642 China; ^2^ College of Forestry and Landscape Architecture South China Agricultural University Guangzhou 510642 China; ^3^ Key Laboratory of National Forestry and Grassland Administration for Orchid Conservation and Utilization at College of Landscape Architecture and Art Fujian Agriculture and Forestry University Fuzhou 350002 China; ^4^ School of Life Sciences Sun Yat‐sen University Guangzhou 510275 China; ^5^ Research Institute of Sun Yat‐sen University in Shenzhen Shenzhen 518107 China

**Keywords:** full‐length transcriptomes, gene expression, motif types, *Paphiopedilum malipoense*, simple sequence repeats (SSRs)

## Abstract

Simple sequence repeats (SSRs) are found in nonrandom distributions in genomes and are thought to impact gene expression. The distribution patterns of 48 295 SSRs of *Paphiopedilum malipoense* are mined and characterized based on the first full‐length transcriptome and comprehensive transcriptome dataset from 12 organs. Statistical genomics analyses are used to investigate how SSRs in transcripts affect gene expression. The results demonstrate the correlations between SSR distributions, characteristics, and expression level. Nine expression‐modulating motifs (expMotifs) are identified and a model is proposed to explain the effect of their key features, potency, and gene function on an intra‐transcribed region scale. The expMotif‐transcribed region combination is the most predominant contributor to the expression‐modulating effect of SSRs, and some intra‐transcribed regions are critical for this effect. Genes containing the same type of expMotif‐SSR elements in the same transcribed region are likely linked in function, regulation, or evolution aspects. This study offers novel evidence to understand how SSRs regulate gene expression and provides potential regulatory elements for plant genetic engineering.

## Graphical abstract

Statistical genomics analyses reveal a high‐density SSR landscape of slipper orchid *P. malipoense* transcriptome. Transcribed SSRs can alter the gene expression potential and tissue‐specificity where they are located. The expression regulation effects of expMotifs are finely delineated, hotspots of expMotifs exist within the transcribed regions, and the mechanisms are illustrated by a model.

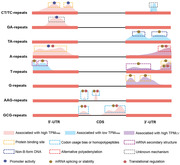

## 1 Introduction

Many repetitive elements are widespread throughout prokaryotic and eukaryotic genomes, and some of them were confirmed to impact gene expression.^[^ ^1^, ^2^, ^3^, ^4^ ^]^ Simple sequence repeats (SSRs), consisting of 1−6 bp units of short, tandemly repeated motifs, are considered an important source of rapid generation of heritable variation and driving the expression evolution and environmental adaptation of species.^[^ ^1^, ^5^ ^]^ The variation in SSRs distributed in genes, transposons, and centromeres can stimulate genomic diversification and promote plant diversity generation and environmental adaptation.^[^ ^6^ ^]^ SSRs within genes could act as “tuning knobs” for regulating gene expression and modulating physiological metabolic processes.^[^ ^6^, ^7^, ^8^ ^]^ Thus, the SSRs were responsible for some of the phenotype changes in plants, including morphogenesis,^[^ ^9^ ^]^ tolerance,^[^ ^10^ ^]^ and reproductive phenology.^[^ ^11^, ^12^ ^]^ SSRs within different transcribed regions can modulate gene expression and functions through various mechanisms. SSR expansion and/or contraction in the CDS region affects the protein product of the corresponding gene and modifies the phenotype directly.^[^ ^6^, ^13^ ^]^ SSR variation in 5’‐UTRs could impact transcription and translation and ultimately modulate gene expression.^[^ ^13^ ^]^ SSR expansion in 3’‐UTRs can cause transcription slippage and lead to gene silencing or a loss of function.^[^ ^13^, ^14^ ^]^ Moreover, SSRs are involved in intra‐ and interspecies gene expression divergence through varieties of motif types, copy numbers, and locations.^[^ ^15^, ^16^, ^17^ ^]^

As indicated by Genome‐wide studies, massive variations of gene expression‐modulating SSRs (eSSRs) within genomes account for a substantial portion of the heritability of complex traits and exhibit evolutionary significance in multiple species.^[^ ^1^, ^18^, ^19^ ^]^ In sunflowers, many functional eSSRs have been identified under selection and facilitating local adaptation across natural populations.^[^ ^16^, ^20^ ^]^ The repeat copy number of SSR in the *Saccharomyces cerevisiae* *SDT1* promoter evolved to the optimal number that achieves the highest gene expression in the experimental evolution setting.^[^ ^21^ ^]^ Understanding the molecular mechanisms underlying SSR‐mediated expression regulation could enhance future research on complex traits and the design of universal regulatory elements for genetic engineering. However, the abundance, hypervariability, and complexity of SSRs in genomes pose challenges in measuring their effect on gene expression systematically based on omic data.

Comprising ≈10% of flowering plant species, orchids (Orchidaceae) possess extraordinarily diverse morphologies and lifestyles and have colonized almost every habitat on Earth.^[^ ^22^, ^23^ ^]^ These features exert their remarkable charisma and make them important models for evolutionary biology and ecology. However, little is known about the composition, distribution, and mechanisms underlying eSSRs in the orchid genome. Orchidaceae has a large and the most variable genome size among angiosperm families (0.33–55.4 pg C^−1^).^[^ ^24^ ^]^ With a genome size range of 4.1–43.1 pg C^−1^, slipper orchids (Cypripedioideae) have some of the largest genomes in orchids, and *Paphiopedilum* exhibits a large genome size of this subfamily (16.5–39.5 pg C^−1^).^[^ ^24^, ^25^ ^]^ Substantial amounts of repetitive sequences play essential roles in genome dynamics, differentiation, and evolution, contributing to the nuclear genome sizes of Cypripedioideae.^[^ ^24^, ^25^ ^]^ We used *P. malipoense* as the research subject to investigate the eSSRs in a large orchid genome with low rates of repetitive DNA removal.^[^ ^26^ ^]^

Herein, we developed a genomic statistics framework to evaluate the expression‐modulating effect of SSRs from omic‐scale data. We adopted joint long‐read SMRT sequencing and short‐read RNA‐Seq, which can resolve reliable repetitive regions, to generate a full‐length reference transcriptome of *P. malipoense*. Further, we identified and characterized the SSRs based on these data. We confirmed the associations between SSR features (including motif types, location, abundance, density, length, etc.) and gene expression levels, identified nine motifs significantly affecting gene expression (abbreviated as expMotif), and finely described the key features, potency, and gene function involving gene expression regulation on an intra‐transcribed region scale. This system‐level study provides new insight into the role of repetitive sequences in the adaptation and evolution of orchids. The novel discovery of expMotifs informed the understanding of the mechanisms underlying the eSSRs, which were valuable potential regulatory elements for plant genetic engineering.

## 2 Results

### 2.1 Full‐Length Transcriptome of *P. Malipoense* Generated by Hybrid Sequencing

In this study, RNA‐seq and PacBio Iso‐Seq were combined to obtain the full‐length transcriptome of *P. malipoense*. A fine sampling of seven flower parts (labellum, petals, dorsal sepals, synsepals, stamen, staminodes, and gynoecia) was employed for RNA‐seq on the Illumina HiSeq 4000 platform. After quality filtering, a total of 57.26 Gb clean data were generated with an average of 25 119 394 clean reads per sample (Data S1, Supporting Information). RNA samples from roots, stems, leaves, petals, sepals, gynandrium, and peduncles were mixed and sequenced using the PacBio Sequel platform. A total of 13.25 Gb of raw data and 5 611 491 subreads were generated, and the average length was 2362 bp (Table S1, Supporting Information). The SMRTlink v5.1 pipeline was used to process the raw data and obtained 360 416 circular consensus sequences (CCS), including 60 627 non‐full length reads and 283 475 full length non‐chimeric reads. After iterative clustering for error correction (ICE) and hybrid error correction using RNA‐seq data, 126 513 high‐quality full‐length transcripts with a mean length of 2792 bp were generated. After removing redundant sequences using CD‐HIT, a total of 63 940 nonredundant transcripts (unigenes) were finally obtained. All unigenes were functionally annotated by searching seven public databases, including NR, SwissProt, eggNOG, KOG, GO, KEGG, and Pfam. A total of 55 116 (86.20%) unigenes were annotated, and 24 457 (38.25%) unigenes were annotated in all databases (Table S2, Supporting Information).

### 2.2 Identification of SSRs in the *P. Malipoense* Transcriptome

**Figure 1:**
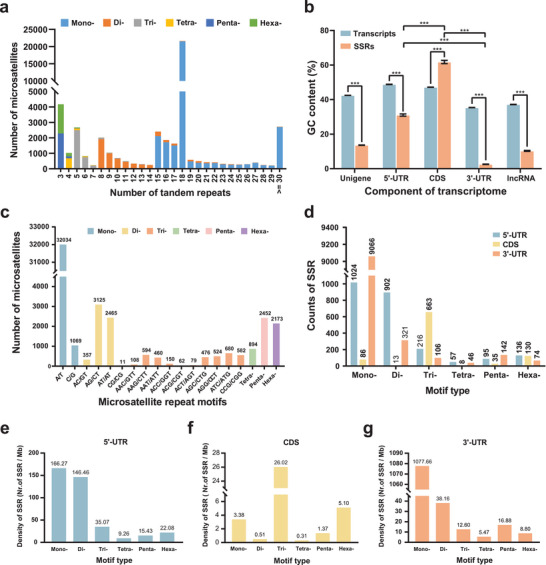
Statistics of SSRs in the *P. malipoense* transcriptome. a) Distribution of tandem repeat number of SSRs in the *P. malipoense* transcriptome. b) GC contents of different components in the *P. malipoense* transcriptome. The error bars show the standard error of the mean. ^***^ indicates *p* < 0.001 (Wilcoxon rank sum test or Dunn's pairwise test). c) Distribution of SSR motif types. d) Counts of six SSR motif sizes in three transcribed regions. e–g) Density (SSR counts per Mb) of six SSR motif sizes in the 5’‐UTR (e), CDS (f), and 3’‐UTR (g).

The MISA program was used to detect potential SSR loci in the *P. malipoense* transcriptome. A total of 48 295 perfect SSRs were identified from 18 770 unigenes, with 7476 unigenes containing more than one SSR. The frequency of SSRs was 75.53%, and the mean density (SSR counts per Mb) was 248.11 (**Table** **1**). A total of 575 SSRs present in compound formation were composed of 1,179 SSRs, and each compound SSR consisted of 2–4 tandem single SSRs. Mono‐nucleotides (33 103, 68.56%) accounted for the vast majority of SSRs in the *P. malipoense* transcriptome, followed by di‐nucleotides (5958, 12.34%), tri‐nucleotides (3715, 7.69%), penta‐nucleotides (2452, 5.08%), hexa‐nucleotides (2173, 4.50%), and tetra‐nucleotides (894, 1.85%) (Table 1). The SSR tract length ranged from 15 to 217 bp, with an average of 19.51 bp. The SSR tract length was significantly different between the motif sizes (Kruskal‒Wallis rank sum test, *χ* ^2^ = 9722.9, df = 5, *p* value < 2.2e−16), and slightly declined with increasing motif size (Kendall rank correlation coefficient tau = −0.1969, *p* value < 2.2e−16) (Figure S1, Supporting Information). The repeat number of motifs varied from 3 to 217 with an average of 15.65 (**Figure** **1**a).

**Table 1:** Statistical results of SSRs in the *P. malipoense* transcriptome.

Item	Number
The total number of sequences examined	63 940
The total size of examined sequences [bp]	194 653 584
Total number of identified SSRs	48 295
Number of SSR‐containing sequences	18 770
Number of sequences containing more than one SSR	7476
Number of SSRs present in compound formation	575
Mono‐nucleotide	33 103
Di‐nucleotide	5958
Tri‐nucleotide	3715
Tetra‐nucleotide	894
Penta‐nucleotide	2452
Hexa‐nucleotide	2173

A total of 313 motif types were detected in the 48 295 SSRs. A/T, with a frequency of 66.33%, was the most dominant among all the motifs. AG/CT (3125, 6.47%) and AT/AT (2465, 5.10%) were the top two dinucleotide motifs. The most abundant motif types in the trinucleotide were ATC/ATG (680, 1.41%) and AAG/CTT (594, 1.23%). For tetranucleotides and pentanucleotides, AAAT/ATTT (452, 0.94%) and AAAAT/ATTTT (268, 0.55%) were the most frequent motif types, respectively. Moreover, AAGTGC/ACTTGC (268, 0.55%) and ACCTGC/AGGTGC (260, 0.54%) comprised 24.30% of all hexa‐nucleotides (Figure 1c).

### 2.3 SSR Distribution in Different Transcribed Regions

We utilized ANGEL 3.0 (https://github.com/PacificBiosciences/ANGEL) to predict ORFs, and 15 836 authentic ORFs were verified by BLAST searches against the Nr and SwissProt databases and corrected manually. Noncoding RNAs were identified by employing five analysis methods, including Pfam, CPC2, CNCI, CPAT, and PLEK. After filtering transcripts < 200 bp in length, a total of 8128 ncRNAs were confirmed as lncRNAs by at least four methods. We compared the SSR distribution characteristics among different transcriptome components. Of the 48 295 SSRs, 935, 2430, 9755, and 6281 were located in the CDS, 5’‐UTR, 3’‐UTR, and lncRNA, respectively (**Table** **2**). The highest density of SSRs was observed in the 3’‐UTR, with an average of 1159.56 per Mb, followed by the 5’‐UTR (394.57/Mb) and lncRNAs (265.34/Mb). CDS had the lowest density of SSRs (36.70/Mb). After excluding 28 895 SSRs located in the boundary of CDS and UTRs or lacking solid localization information, 19 401 SSRs were used for subsequent analysis.

**Table 2:** SSR distribution among different transcriptome components.

Region	Number of sequences	Total number of base pairs [bp]	Number of SSR	Density of SSRs [/Mbp]
CDS	15 836	25 475 917	935	36.70
UTRs	31 458	14 571 206	12 185	836.24
5'UTR	15 654	6 158 541	2430	394.57
3'UTR	15 804	8 412 665	9755	1159.56
5'UTR‐CDS boundary	–	–	21	–
CDS‐3'UTR boundary	–	–	1	–
lncRNA	8 128	23 671 941	6281	265.34
Other transcripts	23 474	76 716 792	28 872	376.35

SSR counts in different transcribed regions exhibited significant differences across the motif sizes (Pearson's chi‐square test, *χ* ^2^ = 10058, df = 10, *p* value < 2.2e−16) (Figure 1d). CDS had the lowest SSR frequency and was dominated by trinucleotides (70.91%) (Figure 1f). SSRs in the 5’‐UTR were mostly mono‐nucleotide (42.14%) and di‐nucleotide (37.12%) (Figure 1e). The motif sizes of SSRs were most heterogeneous in the 3’‐UTR with mono‐nucleotide predominance (92.94%), whose SSR density was 31‐fold higher than that of CDS (Figure 1g). Furthermore, the SSR tract length showed a significant difference among genic regions (Kruskal‒Wallis rank sum test, *χ* ^2^ = 668.56, df = 2, *p* value < 2.2e−16). The average length of SSR tracts located in CDS (17.52 bp) was shorter than that in the UTRs, and SSRs in the 3’UTR (19.87 bp) were significantly longer than those in the 5’‐UTR (19.55 bp) (Figure S2, Supporting Information). The Kruskal‒Wallis rank sum test confirmed that the interaction between regions and motif sizes affected the length of SSR tracts (H = 38.09, df = 10, *p* value = 3.66e−05), especially for mononucleotides, dinucleotides, and hexanucleotides (Figure S3, Supporting Information).

We also calculated the GC content of the SSRs and their background sequences. The GC% of SSRs in all of the transcriptome components showed significant differences from the sequences in which they were located (Wilcoxon rank sum test, W = 9971507, *p* value < 2.2e−16) and was lower than the GC% of their background sequences excluding CDS. CDS had the highest GC% of SSRs at 61.78 ± 29.14%, followed by 5’‐UTR (31.06 ± 31.06%) and lncRNA (2.55 ± 12.82%), and 3’‐UTR had the lowest GC% of SSRs. The post hoc test indicated significant differences among all the transcribed regions (Dunn's pairwise test, *p* value < 2.2e−16) (Figure 1b).

Adjusted standardized residuals from the chi‐squared test revealed preferences of motif types among transcribed regions. A/T repeats were significantly enriched within the 3’‐UTR, while C/G repeats were overrepresented in the 5’‐UTR and CDS (*χ* ^2^ = 865.296, df = 2, *p* value = 1.26e−188) (Figure S4, Supporting Information). Di‐nucleotides can be standardized into four types, AT/AT, AC/GT, AG/CT, and CG/CG. There was only one CG/CG repeat detected in the 5’‐UTR, and it was absent from the remaining transcribed regions. AC/GT, AG/CT, and AT/AT repeats were enriched in CDS, 5’‐UTR, and 3’‐UTR, respectively (*χ* ^2^ = 392.04, df = 4, *p* value = 1.44e−81) (Figure S5, Supporting Information). Most trinucleotide motif types were enriched in CDS, excluding AAG/CTT and ACG/CGT, which were dominant in the 5’‐UTR, and AAT/ATT and ACC/GGT, which were overrepresented in the 3’‐UTR (*χ* ^2^ = 246.48, df = 18, *p* value = 4.24e−42) (Figure S6, Supporting Information). Sample sizes of SSRs with longer motif types were too small for residual analysis and were skipped.

### 2.4 Association Between Expression Level and SSR Characteristics

**Figure 2:**
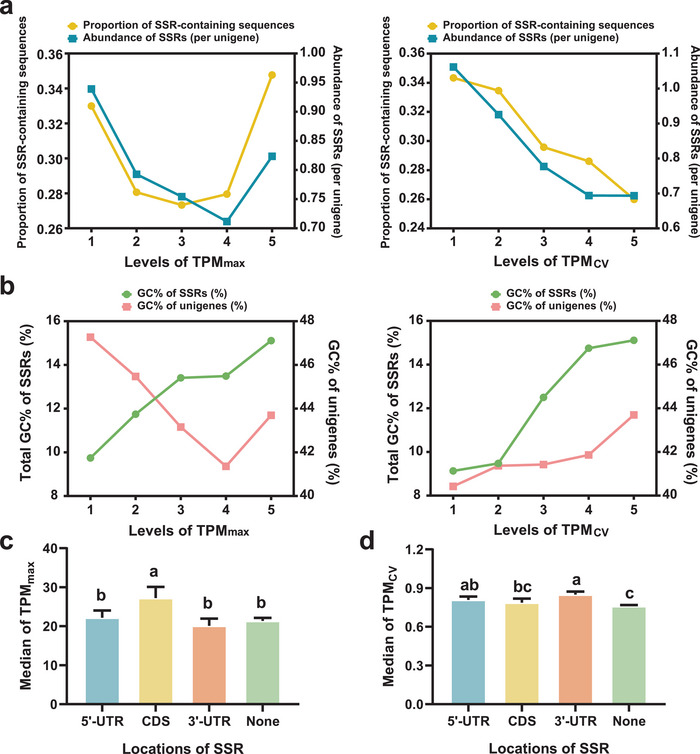
SSR characteristics and location of unigenes with different expression levels. a) Trends of the proportion of SSR‐containing sequences and SSR abundance (SSR counts per unigene) as TPM_max_ (left) and TPM_CV_ (right) decreased. b) Trends of the GC contents of SSRs and their sequence contexts as TPM_max_ (left) and TPM_CV_ (right) decrease. c,d) TPM_max_ (c) and TPM_CV_ (d) of unigenes with SSRs in the different genic regions. The error bar indicates 95% CI. Different letters represent significant differences, and the same letters represent no significant difference (one‐sided test, BH adjusted *p* value < 0.05).

The transcripts per million (TPM) values of all unigenes of the *P. malipoense* transcriptome were calculated for 12 tissues, and the maximum TPM values (TPM_max_) were calculated to represent the expression capacity of each unigene. The coefficient of variation of TPM values (TPM_CV_) was used to characterize the tissue‐specific expression of unigenes. Each variable was divided into five levels, the thresholds for grouping TPM_max_ were set to 1, 10, 100, and 1000, and those for grouping TPM_CV_ were set to 0.5, 1, 1.5, and 3.4641. The threshold of level 1 for TPM_CV_ was set as 3.4641, which represented the unigenes expressed in only one organ. Level 1 indicated the highest values and level 5 indicated the opposite. The number of unigenes in TPM_max_ levels 1−5 were 394, 3264, 16 520, 24 215, and 15 429, respectively (Data S2, Supporting Information). The proportion of SSR‐containing unigenes ranged from 27.33% (level 3) to 34.77% (level 5) and showed a U‐shaped curve relationship with TPM_max_. The SSR abundance (SSR counts per unigene)‐TPM_max_ association also appeared as a U‐shaped curve, as the highest SSR abundance was 0.939 per unigene in level 1, and the lowest value was 0.712 per unigene in level 4 (**Figure** **2**a). There were 1 229, 8 933, 15 865, 30 841, and 2 951 unigenes in levels 1−5 of TPM_CV_ (Data S2, Supporting Information). The abundance of SSRs and proportion of SSR‐containing sequences increased monotonically with TPM_CV_ and ranged from 0.69 to 1.06 per unigene and 26.00−34.34%, respectively (Figure 2a). The global GC content of SSRs showed a negative association with both TPM_max_ and TPM_CV_ levels and was independent of the background GC content (Figure 2b).

We compared four characteristics of SSRs (abundance, density, GC content, and length) using the Kruskal‒Wallis test, and all the characteristics showed significant differences (*p* value < 0.05) among both TPM_max_ and TPM_CV_ levels (Figure S7a–d, Supporting Information). Dunn's post hoc test demonstrated over 50% pairwise differences in SSR density and GC content among different TPM_max_ and TPM_CV_ levels (*p* value < 0.05). The density of SSRs appeared to be positively associated with TPM_max_ (Figure S7a, Supporting Information), while a U‐shaped curve relationship was shown during the decrease in TPM_CV_ (Figure S7a, Supporting Information). The average GC contents of SSRs in each unigenes show similar trends to the global GC content of SSRs (Figure S7b, Supporting Information).

Unsurprisingly, mononucleotides were the majority within all TPM_max_ and TPM_CV_ levels (64.37−82.7%, 65.0−75.5%). The proportion of mononucleotides in SSRs declined with TPM_max_ and TPM_CV_ (Figure S8a, Supporting Information). The expectation of abundance of di‐, tetra‐, and hexa‐nucleotide showed a monotonic increase as TPM_max_ declined (Figure S8b, Supporting Information), and the proportion of SSR‐containing sequences of di‐ and hexa‐nucleotide showed the same trends (Figure S8c, Supporting Information). The abundance of dinucleotides presented a slightly positive association with TPM_CV_ (Figure S8b, Supporting Information), while the proportion of SSR‐containing sequences of dinucleotides showed a different tendency, with the peak value appearing at level 4 (Figure S8c, Supporting Information). These results demonstrated a significant association between SSR distribution and gene expression level as well as tissue specificity of gene expression.

The same analyses were performed on 7229 expressed lncRNAs. The counts of lncRNAs in TPM_max_ levels 1−5 were 27, 87, 507, 2422, and 4166; 385, 1597, 2585, 2642, and 20 lncRNAs in level 1−5 of TPM_CV_. Like unigenes, the abundance of SSRs in lncRNAs increased monotonically with TPM_CV_ and ranged from 0.45 to 0.91 per lncRNA (Figure S9a, Supporting Information). There was no obvious pattern of the proportion of SSR‐containing sequences, abundance, and distribution of GC content of SSRs for different TPM_max_ levels (Figure S9a–d, Supporting Information). The global GC content of SSR of TPM_max_ and TPM_CV_ levels were independent of the contexts (Figure S9b, Supporting Information). Significant differences were found in density, abundance, and GC content of SSRs among TPM_max_ and TPM_CV_ levels (Figure S10, Supporting Information). SSR density and abundance were positively associated with TPM_CV_, and the GC content of SSR showed an opposite trend. In contrast, none of the characteristics showed a monotonical trend during the TPM_max_ increase.

### 2.5 Differential Expression Between SSRs in UTRs and CDS

A total of 12 881 SSRs were found in 15 564 unigenes whose TPM value was greater than 0.01 in all samples. The Kruskal‒Wallis test was utilized to compare TPM_max_ and TPM_CV_ among the unigenes with SSRs distributed in the 5’‐UTR, CDS, and 3’‐UTR and without SSR distribution. Unigenes with SSRs located in more than one region were excluded. Unigenes that have SSRs in different transcribed regions showed significant differences in both TPM_max_ (*χ* ^2^ = 19.925, df = 3, *p* value = 0.00018) and TPM_CV_ (*χ* ^2^ = 94.923, df = 3, *p* value < 2.2e−16). Dunn's pairwise post hoc test demonstrated that the unigenes with SSRs in CDS had a significantly higher TPM_max_ than other groups (Figure 2c), while the appearance of SSRs was associated with higher TPM_CV_, especially in UTRs (Figure 2d). The same statistical tests were performed on different repeat types and motif types. All the repeat types except tetra‐nucleotide showed significant differences in TPM_max_ among locations. Unigenes with mono‐nucleotides in the 5’‐UTR and CDS and penta‐ and hexa‐nucleotides in UTRs tended to have a lower TPM_max_, whereas those with dinucleotides in the 5’‐UTR and tri‐nucleotides located in CDS were associated with a greater TPM_max_ (Table S3, Supporting Information). Unigenes with mono‐, di‐, and pentanucleotides exhibited a significantly different TPM_CV_ among groups, while mononucleotides in CDS, dinucleotides in 3’‐UTR and pentanucleotides in 5’‐UTR were related to a higher TPM_CV_ (Table S4, Supporting Information).

### 2.6 Detection of Putative Expression‐Modulating Motifs

The Kruskal‒Wallis test and Mann‒Whitney test were used to primarily filter the putative expMotifs. Significant heterogeneities of TPM_max_ and TPM_CV_ were detected on unigenes with seven and five fully standardized motifs in the transcribed regions compared with those without these motifs, respectively (*p* value < 0.05) (Tables S5 and S6, Supporting Information). Furthermore, we used linear regression, quadratic regression, cubic regression, and multivariable regression to exploit the relationship between SSR characteristics (abundance, density, length) and the natural log‐transformed TPM_max_ and TPM_CV_ across genic regions. When lnTPM_max_ was set as the response variable, 49 significantly fitted models and 14 optimal models involving nine fully standardized motifs and three genic regions were selected (Table S7 and Figures S11−S13, Supporting Information). All of the bivariate models passed the variance inflation factor (VIF) test, indicating that multicollinearity was not observed. R^2^ of the optimal models, which represents the coefficient of determination was used to measure the predictive power of the model, ranging from 0.026 (A/T, 3’‐UTR) to 0.841 (AGG/CCT, CDS). Moreover, 13 significantly fitted models were obtained when lnTPM_CV_ was the response variable, and four optimal models whose R^2^ ranged from 0.0112 (A/T, 3’‐UTR) to 0.331 (ATC/ATG, 5’UTR) were selected (Table S8 and Figure S14, Supporting Information).

We performed the same analysis for 29 actual motifs corresponding to nine standardized candidates, whose sequence counts were above ten (Table S9, Supporting Information). The results of the Kruskal‒Wallis test or Mann‒Whitney test confirmed that 13 and 6 motifs showed significantly different TPM_max_ and TPM_CV_ values (*p* value < 0.05) among transcribed regions, respectively (Tables S10 and S11, Supporting Information). Moreover, the SSR characteristics of 20 and 10 actual motifs showed significant correlations with lnTPM_max_ and lnTPM_CV_ in regression analyses (R^2^ ranged from 0.023 to 0.928) (Tables S12 and S13, and Figures S15−S17, Supporting Information). In total, 25 (86.21%) actual motifs showed at least one statistical association with expression levels.

The results of the statistical tests and regression analysis provided evidence of the correlation between gene expression level and SSR distribution of protein‐coding genes. Eight of the nine standardized candidate expMotifs were found to significantly associate with gene expression level by the Kruskal‒Wallis test, Mann‒Whitney test, and multiple regression analysis. The A/T and AG/TC repeats in the 5’‐UTR, the A/T, AAG/CTT, AGG/CCT, ATC/ATG, and CCG/CGG repeats in the CDS, and AT/AT and AAAAT/ATTTT repeats in the 3’‐UTR were strongly correlated with TPM_max_. Additionally, AG/CT, AT/AT, and CCG/CGG repeats in the 5'‐UTR and A/T and AG/CT repeats in the 3'‐UTR were strongly associated with TPM_CV._ Furthermore, nine actual expMotifs, corresponding to six standardized candidates, were found to be significant for all the statistical tests. These motifs included T, GA, and CT repeats within the 5'‐UTR; AAG and GCG repeats within the CDS; and A, CT, TC, and TA repeats within the 3'‐UTR, which all had significant correlations with TPM_max_ (Table S9, Supporting Information). Moreover, A repeats in the 5'‐UTR and 3'‐UTR and T and G repeats in the 3'‐UTR were strongly linked to TPM_CV_ (Table S9, Supporting Information).

### 2.7 Relative Position of the Candidate Motif Varies Among Expression Levels

**Figure 3:**
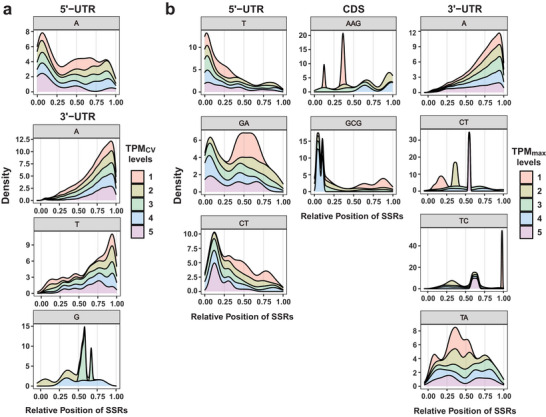
The stacking probability density curves of the relative position of expMotifs within transcribed regions. a) intra‐transcribed region distribution of expMotifs correlated to TPM_CV_ within the 5’‐UTR (top) and 3’‐UTR (bottom). b) Intra‐transcribed region distribution of expMotifs correlated to TPM_max_ within the 5’‐UTR (left), CDS (middle), and 3’‐UTR (right). Different colors indicate different TPM_CV_ and TPM_max_ levels, and the values decrease from level 1 to 5. The ordinate represents probability density, and the abscissa represents the position of SSRs relative to the starting position of the transcribed regions.

The criteria used to define SSRs likely affected SSR characteristics used for regression analysis. To eliminate this influence, probability density curves of the relative position distribution of actual expMotifs within three transcribed regions were plotted. Noticeable discrepancies can be seen in the distribution patterns of almost all of the expMotifs among different TPM_max_ and TPM_CV_ levels (**Figure** **3**). Notably, different actual expMotifs, which corresponded to the same fully expMotif, showed distinct distribution patterns. For instance, the unigenes with the highest TPM_max_ level presented a special probability density peak of GA repeats at 0.45 to 0.62 within the 5’‐UTR, and those with lower expression levels had GA repeat peaks at approximately one‐quarter of the 5’‐UTR. CT repeats appeared to have characteristic peaks at 0.485 and 0.796 within the 5’‐UTR of high TPM_max_ levels, and the peaks at the 5’ terminus were associated with lower TPM_max_ levels. The peaks of CT repeats within the 3’‐UTR of TPM_max_ levels 1, 2, and 5 were 0.171, 0.362, and 0.548, and those of TC repeats were 0.980, 0.320, and 0.621, respectively. This observation raises the possibility that the regulatory effects of expMotifs on gene expression depend upon the type of actual motifs composed of the SSRs and their positional distribution on the gene.

Exceptionally, the A repeats within the 3’‐UTR tended to be distributed at the 3’ terminus, and no significant difference was found among different TPM_max_ and TPM_CV_ levels, whereas A repeats within the 5’‐UTR and T repeats within the 5’‐UTR and 3’‐UTR showed different density curves. It can thus be suggested that the A repeats within the 3’‐UTR may modulate the gene expression level due to another mechanism, such as an AU‐enriched element (ARE)‐related regulatory mechanism of mRNA stability. We cannot rule out the possible reason for the extremely large sample size of A repeat‐containing sequences within the 3’‐UTR.

### 2.8 Functional Enrichment of Expression‐Modulating Motif‐Containing Unigenes

**Figure 4:**
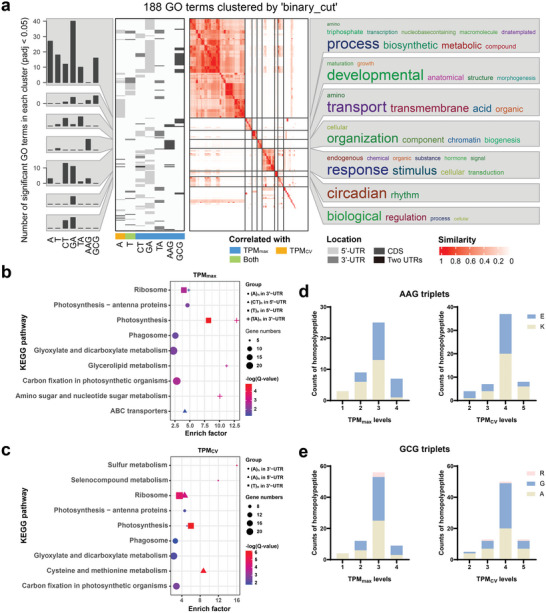
Potential functions of expMotif‐SSRs. a) GO analysis for biological processes of each type of expMotif‐SSR. GO terms representing each cluster were displayed as a word cloud, with the size of the word indicating the frequency of appearance in the terms. b,c) Significantly enriched KEGG pathways of each type of expMotif correlated to TPM_max_ (b) and TPM_CV_ (c). d,e) Predominant amino acids of homopolypeptides coded by AAG repeats (d) and GCG repeats (e) with different TPM_max_ (left) and TPM_CV_ (right).

To better understand the possible function of nine actual expMotifs, unigenes containing each actual expMotif were used as query sets to conduct GO enrichment analysis against the background of all transcripts. The results were compared and summarized in **Figure** **4**a. Excluding the TC repeat with no enriched GO term, a total of 188 GO terms (Q‐value < 0.05) in the biological processes category were enriched and clustered into seven classes. The cluster of biosynthetic processes, including transcription and translation, enriched the largest number of significant GO terms for six expMotifs, except for AAG repeat. The cluster with the second‐highest number of significant GO terms was assigned to the cluster of response to stimuli. The GA repeats within the 5’‐UTR, the A and TA repeats within the 3’‐UTR, the CT repeats within two UTRs, and the AAG repeats within the CDS were associated with this cluster. The GA repeats within the 5’‐UTR and the AAG and GCG repeats within the CDS were also enriched in the anatomical structure development cluster. The organic acid transmembrane transport cluster contained GO terms that were represented primarily by CT repeats within the 5’‐UTR and TA repeats within the 3’‐UTR. The circadian rhythm cluster was uniquely enriched by GA repeats. Moreover, CT and GA repeats located in the 5’‐UTR were related to the biological regulation process.

KEGG pathway enrichment analysis of each expMotif‐containing unigene indicated that 12 pathways were significantly enriched (Q‐value < 0.05), nine of which were assigned to the metabolism class, and six overrepresented pathways were shared by TPM_max_‐ and TPM_CV_‐associated expMotifs (Figure 4b,c). Notably, there were three enriched KEGG pathways related to photosynthesis that involved TA repeats in the 3’‐UTR and A and T repeats in the two UTRs. GO and KEGG pathway enrichment analyses revealed a functional preference for SSRs with different motif types and transcribed regions, even though expMotifs corresponding to the same standardized motif tended to exhibit distinct GO and KEGG enrichment profiles.

### 2.9 Validation of the Regulatory Effect of expMotif‐SSRs

To validate the regulatory effect of expMotif‐SSRs, MYB, F‐box, and TCP protein families, which were essential for plant development and stress adaptation, were selected randomly to compare the expression levels between expMotif‐SSR containing unigenes and their paralogs without expMotif‐SSR.

**Figure 5:**
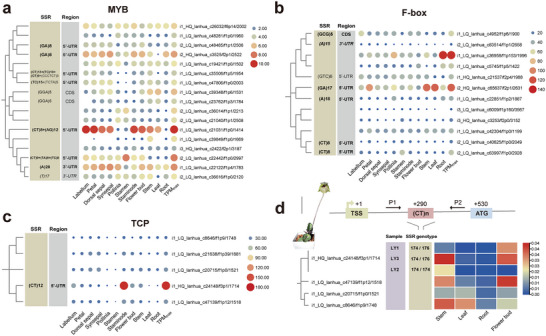
Validation of expression‐modulating effects of expMotif‐SSR. a) Heatmap of TPM values of expMotif‐SSR containing MYB genes and their paralogs. The SSR distribution of all genes was listed on the left. The NJ tree based on unigene sequences depicts the phylogenetic relationships among the genes. b) Heatmap of TPM values of expMotif‐SSR containing F‐box genes and their paralogs. c) Heatmap of TPM values of expMotif‐SSR containing TCP gene and their paralogs. d) The qRTPCR validation of TCP genes. The heatmap shows the 2^−ΔCT^ values. The 2^−ΔCT^ of each sample and their genotype at expMotif‐SSR loci are displayed separately, and the 2^−ΔCT^ of the remaining SSR‐free paralogs are averaged. The schematic representation of expMotif‐SSR in i1_HQ_lanhua_c24148/f3p1/1714 is above the graph. The relative position of CT repeat locus (red, +290), translation start site (ATG, blue, +530) to transcriptional start site (TSS, green, +1) are drawn. Primers P1 and P2 (expected size 177 bp) were used for the genotyping of the expMotif‐SSR locus. The photo shows the morphology of *P. malipoense*. The scale bar represents 10 cm.

Among the three protein families, over half (4/7, 4/6, and 1/1) expMotif‐SSR containing unigenes have TPM_max_ greater than their paralogs without expMotif‐SSR (**Figure** **5**a–c). These unigenes contain expMotif‐SSRs including AG, GA, CT, TC, and TA in 5’‐UTR, GCG repeats in CDS, and A repeat in 3’‐UTR. The A repeat in 3’‐UTR of i2_LQ_lanhua_c83514/f1p1/2658, belonging to the F‐box family, is associated with high TPM_CV_. A total of ten expMotif‐SSR loci (62.5%) showed expression‐modulating effects as predicted.

The expMotif‐SSRs of TCP gene i1_HQ_lanhua_c24148/f3p1/1714 were genotyped by CE. This transcription factor has multiple roles in the regulation of plant development, including chloroplast biogenesis,^[^ ^27^ ^]^ plant stature,^[^ ^28^ ^]^ leaf shape,^[^ ^29^ ^]^ and trichome development in flowers.^[^ ^29^ ^]^ Two alleles differ by one repeat unit (2 bp) were detected in three individuals (Figure 5d; Figure S18, Supporting Information). The genotype of this SSR locus was represented as the amplicon length following the convention, as 174/176 for LY1 and LY3, 174/174 for LY2. Results of quantitative Real‐Time PCR (qRTPCR) showed that the maximum expression level of i1_HQ_lanhua_c24148/f3p1/1714 was much higher in the heterozygous individuals (LY1 and LY3) with the genotype 174/176 than in LY2 with the genotype 174/174 (Figure 5d)_._ The maximum expression level of this gene in LY1 and LY3 was significantly higher than its paralogous i1_LQ_lanhua_c20715/f1p0/1521 and i1_LQ_lanhua_c8646/f1p9/1748, and slightly lower than i1_LQ_lanhua_c47139/f1p12/1518 (Figure 5d; Data S3, Supporting Information). These results indicate that at this expMotif‐SSR locus, a longer allele, even with only one repeat unit difference on one allele, can obviously alter the gene expression where it is located.

## 3 Discussion

Here, we analyzed the SSR composition, features, and correlation with gene expression using comprehensive transcriptome data from *P. malipoense*. We found a high‐density transcriptome SSR landscape in this large genome orchid. SSR type and features were heterogeneous among the transcriptome components. We confirmed the relationship between SSR type and features with the expression capacity and the tissue‐specific expression. The motif type and transcribed region combination are the most predominant contributors to the expression‐modulating effect of SSRs. The abundance, density, or SSR tract length of expMotif‐SSR has a subtle but significant effect on gene expression. We identified nine expMotifs and revealed their acting transcribed regions and potential functions. Some intra‐transcribed regions were recognized as expMotif‐SSR hotspots, which can guide further identified microsatellite loci containing the expMotifs may function as “tuning knobs” of gene expression regulation. Our study not only reveals the landscape of transcriptome SSR and their role in expression regulation, but also provides a feasible, generic, and high‐throughput method to investigate expression‐modulating SSR loci across species.

Generally, the differences in density, frequency, and length of SSRs across species are dependent on the size of the unigene assembly dataset, sequence redundancy, SSR definition criteria, and genome organization. The pipeline we used to mine SSRs from full‐length transcriptome data can minimize the error from assembly and sequence redundancy. In this study, we identified 48 295 SSRs from 18 770 unigenes of the *P. malipoense* transcriptome, with a frequency of 75.53% and a density of one SSR per 4.03 kb. It was much higher than that of other species of Orchidaceae, such as *Paphiopedilum hirsutissimum* (1/5.42 kb),^[^ ^30^ ^]^ *Phalaenopsis aphrodite* subsp. *formosana* (1/20.22 kb),^[^ ^31^ ^]^ *Dendrobium officinale* (1/21.83 kb),^[^ ^32^ ^]^ *Cymbidium ensifolium* (1/17.56 kb),^[^ ^33^ ^]^ and *Bletilla striata* (1/4.41 kb),^[^ ^34^ ^]^ supported the low rates of repetitive DNA removal are driving the genome evolution in this group. SSRs are nonrandomly distributed in different transcriptome components. 3’‐UTRs have the highest SSR density (1/0.86 kb), followed by 5’‐UTRs (1/2.53 kb) and CDS (1/27.25 kb). The length of SSRs within CDS was significantly shorter than that of UTRs, and 3’‐UTRs had the highest average length of SSR tracts. This implies a weaker evolutionary constraint of SSRs in UTRs, especially in the 3’‐UTR, which is consistent with reports from the human genome.^[^ ^35^ ^]^ As in most plant species, SSRs of *P. malipoense* located within CDS were dominated by trinucleotides.^[^ ^6^, ^36^ ^]^ CDS regions have a lower density and length of SSR tracts due to stronger selection pressure, especially against frameshift mutation.^[^ ^6^ ^]^ Indels of trinucleotide within CDS will not cause a frameshift and undergo less strict constraints, therefore showing no significant difference in density and length among transcribed regions. SSRs in UTRs are subject to lower evolutionary constraints and exhibit a longer length, while different motif compositions and lengths between UTRs suggest an influence of context.

The SSR features were linked to gene expression. The abundance, the proportion of SSR‐containing sequences, and GC%, average length, and density of SSRs were significantly different among TPM_max_ and TPM_CV_ levels, which measured the maximum expression capacity and the tissue‐specific expression level. Notably, the SSR density appeared to have a strong monotonically positive association with TPM_max_, and the relationship between GC% and TPM_CV_ was the opposite. This indicates that various aspects of gene expression are strongly associated with certain SSR features. The transcribed region was another key factor influencing the effect of SSRs on gene expression. The presence of SSRs in CDS was relative to high TPM_max_. SSRs within CDS are usually considered adverse for eukaryotes due to repeat instability changing the composition of mRNA or protein and leading to gain or loss of gene function, especially for human cancers and other diseases.^[^ ^13^ ^]^ Thus, the emergence and expansion of SSRs within CDS should be constrained strictly. However, Ranathunge et al. reported an enrichment of gene expression correlated SSRs in the CDS of sunflowers.^[^ ^16^ ^]^ Our results provide novel evidence to show that coding region SSRs may facilitate gene expression and have potential functions, although their mechanisms for expression regulation remain elusive. Some trinucleotide repeats, including those in CDS, were suggested to impact nucleosome binding and modulate transcription rates.^[^ ^37^ ^]^ SSRs within UTRs, particularly in the 3’‐UTR, were associated with high TPM_CV_. This implies that SSRs in UTRs are critical for higher specific regulation of gene expression, which may serve as cis‐regulatory elements involved in chromatin state modifications, transcription factor binding,^[^ ^8^ ^]^ mRNA turnover,^[^ ^38^ ^]^ microRNA interactions,^[^ ^6^ ^]^ antisense transcription,^[^ ^39^ ^]^ etc. The GC content of the SSRs showed significant differences among the expression level and transcribed regions and was independent of context. Intriguingly, the AT‐rich SSRs appeared simultaneously with a large length and density of SSRs. Longer SSRs preferred a higher mutation rate,^[^ ^40^ ^]^ and the GC% has been shown to have a notable impact on the probability of mutations.^[^ ^41^, ^42^ ^]^ This phenomenon implied that SSRs underwent district evolutionary dynamics that were context‐independent at the intra‐transcribed region scale and probably had diverse functions.

We detected the association of standardized and actual motif types on gene expression, and the effects of motif type, transcribed region, SSR characteristics, and their interaction were determined. Eight and nine standardized and actual motifs were identified as expMotifs with 13 and 11 statistically significant motif‐region combinations. Notably, these expMotifs were highly coincident with the high‐abundance motifs enriched in three transcribed regions where they act as regulatory elements. All the expMotifs are smaller motif sizes (mono‐, di‐, and trinucleotides), which are more prevalent in the *P. malipoense* transcriptome, as is commonly reported in other angiosperms.^[^ ^15^, ^31^, ^32^, ^33^, ^34^ ^]^ AG/CT and AT/AT, the primary sources of dinucleotide expMotifs in the 5’‐UTR and 3’‐UTR, are widespread in most plant species, especially in UTRs.^[^ ^15^ ^]^ The AAG/CTT and CCG/CGG, acting as expMotifs in CDS, are frequent trinucleotides in *P. malipoense* and other orchid species.^[^ ^31^, ^32^, ^33^, ^34^ ^]^ This phenomenon hints at a general role for these expMotif‐SSRs in gene expression across plant species.

In previous studies, all the overlapping and complementary actual motifs were usually integrated into a fully standardized motif and used for statistical expression‐related analyses,^[^ ^6^, ^15^, ^16^ ^]^ while most experimental studies focused on specific actual SSR loci.^[^ ^8^, ^39^ ^]^ Interestingly, we found that some of the different actual expMotifs, which corresponded to the same fully standardized motif, had different statistical tests and modeling results and showed distinct distribution patterns. This implied that for at least some of the expMotifs, their expression‐modulating effect is sensitive to the actual motif type and has strand specificity. This was also likely the reason why past in silico research did not provide very consistent conclusions with each other and with experimental research. This phenomenon points to various mechanisms underlying expMotif‐SSRs. Compared with UTRs, triplets within CDS are in favor of an actual motif‐dependent expression modulation effect due to any mutation, even to the overlapping motif, which would probably lead to changes in amino acid sequence or codon usage. A total of 78.95% and 83.72% of the optimal models obtained from regression analysis were based on SSR density. This result points to the critical role of density in the expression modulation of the effect of expMotif. At the same time, the SSR abundance and length may be affected by criteria used for SSR definition and reducing the prediction power of these characteristics. The probability density distribution of each type of expMotif‐SSR locates the expMotif‐SSRs on an intra‐transcribed region scale and presents great variation among TPM levels and motif types. This adds further evidence that expMotif‐SSRs and their distribution patterns are likely involved in the regulation of gene expression and have diverse mechanisms.

**Figure 6:**
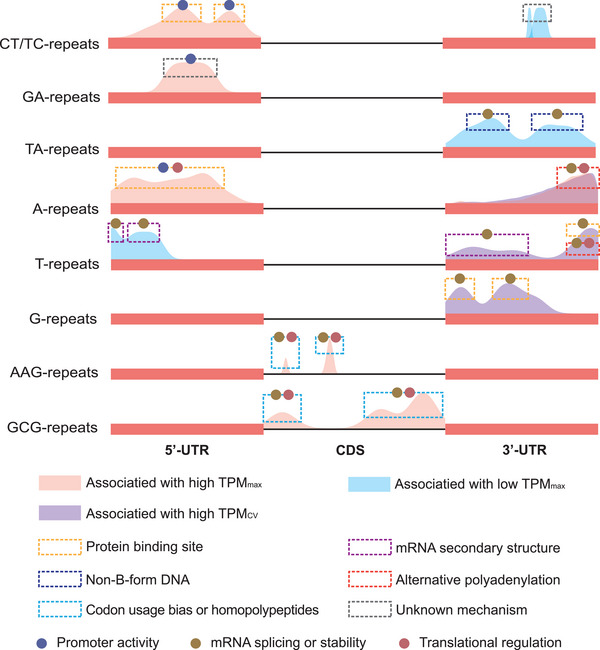
An intra‐transcribed region scale model to explain the expression regulatory effects of expMotif‐SSRs according to our findings and previous experimental studies. Probability density curves show distribution peaks of each expMotif (from Figure 3). The dashed‐line boxes indicate potential regulatory mechanisms of expMotif‐SSRs in corresponding peaks. Dots represent processes of gene expression impacted by expMotif‐SSRs.

Experimental evidence supported the expression regulation effect on some expMotifs identified in our study. A positive selection of AG/CT and AAG/CTT in the 5’‐UTR upstream in *Arabidopsis* ^[^ ^43^ ^]^ and *Phalaenopsis* ^[^ ^31^ ^]^ played significant roles in modulating gene expression and translation.^[^ ^44^, ^45^ ^]^ AT‐rich motifs within the 3’‐UTR were highly overrepresented due to certain cis‐elements, such as the AU‐rich elements (AREs), which regulate mRNA stability.^[^ ^38^ ^]^ Multiple members of the heterogeneous nuclear ribonucleoprotein (hnRNP) family of proteins are known to bind to G‐rich RNA sequences^[^ ^46^, ^47^, ^48^ ^]^ indicating a common regulatory function of these sequences in 3’‐UTR.^[^ ^49^ ^]^ The correlation between AAG or GCG repeats and mRNA levels may be due to codon usage bias, which has verified a positive correlation with expression levels.^[^ ^50^ ^]^ The dominant amino acids coded by AAG and GCG repeats and their proportion ebb and flow as TPM_max_ increases in the *P. malipoense* transcriptome but not for TPM_CV_ (Figure 4d,e) implying that the expression regulation effect of triplet repeats within CDS was not tissue‐specific and may be linked with codon usage or features of homopolypeptides. The alternative polyadenylation (APA), a universal mechanism of posttranscriptional regulation,^[^ ^51^ ^]^ could explain the correlation between A repeats in the 3’‐UTR and high expression potential and tissue‐specificity. The A repeats in 5’‐UTR may act as a protein binding site to regulate promoter activity and translation.^[^ ^52^, ^53^, ^54^ ^]^ Therefore, we propose a model summarizing our findings and previous experimental studies (**Figure** **6**).

Functional enrichment showed different results across expMotif‐transcribed region combinations, implying that genes containing the same expMotif‐SSR element are functionally, regulatorily, and evolutionarily linked, similar to multiple cases of T repeat instability involved in human cancers.^[^ ^55^, ^56^, ^57^, ^58^ ^]^ The mechanism and biological significance of this phenomenon requires further investigation.

The expression level comparisons of MYB, F‐box, and TCP family paralogs demonstrated that our approach for predicting eSSR is effective, but not all expMotif‐SSRs are eSSRs. The gene with different expMotif‐SSR tract lengths presents distinct expression levels and patterns, even if only one allele differs by one repeat unit. The variation of expMotif‐SSR loci among samples used for RNA‐seq and qRTPCR can also explain the subtle differences between the expression patterns of the TCP family obtained from the two experiments. Our results confirmed that an eSSR locus can exhibit a continuous allele spectrum, allowing it to act as a tuning knob to adjust gene expression. We used individuals from different cloned lines for sequencing and experimental validation, yet, we still cannot guarantee to obtain the entire SSR allele spectrums of this species. In the future, large sample size studies in a phylogenetic context combined with a more refined, targeted, and locus‐specific experimental validation approach will help identify evolutionary significant eSSRs and uncover the mechanisms behind our findings.

## 4 Conclusion

The current study reports a high‐density SSR landscape in *P. malipoense* transcriptome. and provide new insights into the role of SSRs in orchid expression evolution and environmental adaptability. The fine exploration of the key features, potency, and gene function of expMotifs helps to understand the mechanisms underlying SSR‐relative expression regulation and may guide the development of universal regulatory elements for plant genetic engineering. We also develop a pipeline generally applicable to studying repetitive elements involved in expression regulation.

## 5 Experimental Section

### Plant Sampling and RNA Extraction

The plant materials for this experiment were collected from the cultivated plant of *P. malipoense* (specimen code Z.J. Liu 7314) in the Orchid Garden of the Key Laboratory of National Forestry and Grassland Administration for Orchid Conservation and Utilization. Whole plants of *P. malipoense* were divided into roots, stems, leaves, petals, sepals, gynandria, and peduncles and collected for full‐length transcriptome sequencing. Meanwhile, seven samples were collected from different flower organs (labella, petals, synsepals, dorsal sepals, stamens, staminodes, and gynoecia) separately for organ‐specific next generation sequencing. All the samples were collected from mature organs. The samples used in the study represent the same cloned line. After harvesting, the tissues were placed in liquid nitrogen for RNA extraction. All tissues were ground on dry ice, and total RNA was isolated using a Tiangen RNAprep Pure Plant Kit (Tiangen, Beijing, China). The same amount of RNA from each tissue was mixed for full‐length sequencing. RNA degradation and contamination were monitored on 1% agarose gels and were measured using a Qubit RNA Assay Kit in a Qubit 2.0 Fluorometer (Life Technologies, CA, USA). RNA purity was checked using a NanoPhotometer spectrophotometer (IMPLEN, CA, USA). The RNA Nano 6000 Assay Kit of the Agilent Bioanalyzer 2100 system (Agilent Technologies, CA, USA) was used to assess RNA integrity.

### Illumina Library Preparation and Sequencing

Illumina sequencing libraries were generated using the NEBNext UltraTM RNA Library Prep Kit for Illumina (NEB, USA) following the manufacturer's recommendations, and index codes were added to attribute sequences to each sample. The library preparations were sequenced on an Illumina HiSeq 4000 platform, and 150 bp paired‐end reads were generated. The average count of clean reads for each sample was 25119394.43. After sequencing, raw reads were filtered to remove the adaptor sequences and low‐quality reads. The remaining high‐quality clean data were used for organ‐specific expression analysis.

### PacBio Library Preparation, Sequencing, and Processing

The Iso‐Seq (Isoform Sequencing) library was prepared according to the Iso‐Seq protocol using the Clontech SMARTer PCR cDNA Synthesis Kit and the BluePippin Size Selection System protocol described by Pacific Biosciences (PN 100‐092‐800‐03). Single‐molecule real‐time (SMRT) sequencing was performed on a PacBio Sequel platform (Pacific Biosciences, CA, USA).

The raw data were processed using SMRTlink 5.1 software (http://www.pacb.com/products‐and‐services/analytical‐sofware/smrt‐analysis/). CCSs were generated from subread BAM files and were then classified into full‐length and nonfull‐length reads. Nonfull‐length and full‐length FASTA files were then fed into the clustering step using isoform‐level clustering (ICE), followed by initial Arrow polishing. Additional nucleotide errors in consensus reads were corrected using Illumina RNAseq data with the software LoRDEC as described by Hoang et al.^[^ ^59^ ^]^ CD‐HIT (cd‐hit‐est with default options) was used to reduce the redundancy in consensus corrected reads^[^ ^60^ ^]^ and finally obtained the unigenes as the reference transcriptome for further analyses.

### Functional Annotation and Expression Level Estimation of Transcripts

Functional annotations of the reference transcriptome were conducted using the BLAST program^[^ ^61^ ^]^ (E‐value < 1× 10^−5^) against NR (NCBI nonredundant protein sequences) and Swiss–Prot (a manually annotated and reviewed protein sequence database). Hmmscan software (http://hmmer.org/) was used for Pfam (Protein family) database analysis. In addition, eggNOG‐mapper^[^ ^62^ ^]^ was utilized for eggNOG (Evolutionary genealogy of genes: nonsupervised orthologous groups), KOG (Clusters of eukaryotic orthologous groups), and GO (Gene Ontology) annotation. KEGG (Kyoto Encyclopedia of Genes and Genomes) prediction was performed by KAAS (KEGG automatic annotation server) (E‐value < 1× 10^−10^).^[^ ^63^ ^]^ A high‐quality prediction of coding DNA sequence (CDS) regions was generated via ANGEL pipeline (https://github.com/PacificBiosciences/ANGEL) and BLAST against NR and Swiss–Prot database (E‐value < 1× 10^−5^). Five analysis methods were employed to predict the coding potential of transcripts, including Pfam^[^ ^64^ ^]^ (‐e_seq 0.001, ‐e_dom 0.001, ‐translate, ‐as), CPC2 (Coding potential calculator version 2, with default parameters),^[^ ^65^ ^]^ CNCI (Coding‐noncoding index),^[^ ^66^ ^]^ CPAT (Coding‐potential assessment tool; ‐s 0, ‐m pl, ‐l 200, ‐e 2),^[^ ^67^ ^]^ and PLEK (predictor of long noncoding RNAs and messenger RNAs based on k‐mer scheme; ‐minlength 200).^[^ ^68^ ^]^ Unigenes predicted without coding potential by at least four tools mentioned above and longer than 200 bp were identified as long noncoding RNAs (lncRNAs). The confident protein sequences from Orchidaceae species were used for ANGEL training. All potential noncoding genes and *de novo* predicted CDSs without BLAST hits were removed from the CDS dataset strictly to confirm that only high‐confident prediction was used for subsequent analyses.

An RNA‐seq‐based transcriptome dataset was constructed using 12 organ transcriptomes covered almost the whole plant: seven of them were sequenced in this study and the remaining five transcriptomes were sampled from the same cloned line and published previously (including roots, stems, pollinia, leaves, and flower buds; SRA accession numbers SRX2938661, SRX2938647, SRX2938646, SRX2938660, and SRX2938639). RSEM (https://github.com/deweylab/RSEM) was used to map this dataset to the full‐length reference transcriptome and quantify read counts. The transcripts per million (TPM) values of all unigenes were calculated for each organ. The maximum TPM (TPM_max_) of each unigene was considered to represent the expression capacity of this unigene, and the coefficient of variation for TPM (TPM_CV_) was used to measure the tissue‐specific expression patterns for each unigene. All unigenes were used into groups according to TPM_max_ and TPM_CV_. The TPM_max_ and TPM_CV_ levels were labeled according to the criteria outlined in Table S14 and Data S3 (Supporting Information).

### SSR Identification and Characterization

The SSRs in the reference transcriptome of *P. malipoense* were identified using MISA (http://pgrc.ipkgatersleben.de/misa/misa.html). The criteria for the SSR search were set as follows: mono‐, di‐, tri‐, tetra‐, penta‐, and hexa‐ with minimum repeat units of 15, 8, 5, 4, 3, and 3, respectively. Two or more adjacent SSRs without interrupted bases were defined as compound SSRs. The genic region distribution of SSRs was inferred from the start and end positions of previously predicted CDS using custom Perl scripts (the Perl scripts were deposited in the GitHub at https://github.com/yy‐liang/SSR_Mapping). Chi‐square tests were conducted to test whether significant differences existed in SSR prevalence among different genic regions (5’‐UTR, CDS, and 3’‐UTR). The adjusted standardized residuals were reported from the chi‐squared test showing the SSR distribution deviations from neutral expectations. All statistical analyses were completed in R version 4.2.2 (https://www.r‐project.org/). The Kruskal‒Wallis rank sum test^[^ ^69^ ^]^ and Wilcoxon rank sum test were implemented for all transcripts to test the effect of motif size and genic region on SSR tract length and GC content. Furthermore, SSRs were grouped in the genic region and expression level (TPM_max_ and TPM_CV_, described previously). The SSR characteristics of each group were calculated, including abundance, density, GC content, and SSR tract length. The same tests were performed for all transcripts and lncRNAs respectively, to investigate the influence of SSR distribution on TPM_max_ and TPM_CV_.

### Statistical Analyses of SSRs and Expression Level

The Kruskal‒Wallis test and Mann‒Whitney test (*p* value < 0.05) were used to primarily filter the putative expMotifs, and the motifs with significant results were identified as candidates. Dunn's post hoc test was used to detect the interaction between motif types and genic location (UTRs or CDS) of candidates at the expression level. Furthermore, linear, quadratic, cubic, and multivariable regressions were used to assess the association between expMotif‐SSR characteristics (abundance, density, length) and the natural log‐transformed TPM_max_ and TPM_CV_ across genic regions. The best‐fitting model was selected according to the Bayesian information criterion (BIC), and the overfitting model was excluded manually. Integrating the results of statistical tests and regression analyses, only the motifs that showed significant results were identified as final expMotifs. In addition, the relative position of expMotif‐SSRs to the transcribed region where they are located was calculated base by base using the following equation: relative position = (actual position of SSR − start position of transcribed region + 1) / transcribed region length. Probability density curves were generated using ggplot2^[^ ^70^ ^]^ by TPM_max_ and TPM_CV_ subgroup.

### GO and KEGG Enrichment Analyses

To infer the possible function across expMotifs in each genic region, GO and KEGG enrichment analyses were performed utilizing hypergeometric tests with Tbtools.^[^ ^71^ ^]^ The enrichment *p* values were adjusted using the Benjamin and Hochberg correction, and the threshold for significant enrichment was a Q‐value < 0.05. The R package simplifyEnrichment^[^ ^72^ ^]^ was used to cluster and visualize the GO enrichment results based on the similarity of GO terms by the binary cut method.

### Experimental Validation

To validate the expression‐modulating effect of expMotif‐SSRs, the expression levels between expMotif‐SSR containing unigenes and their paralogs without expMotif‐SSR were compared. 14 expMotif‐SSRs containing unigenes belonging to three protein families (MYB, F‐box, and TCP families) were randomly selected, which were essential for plant development and stress adaptation. The paralogous protein‐coding unigenes were obtained by the BLASTP compare program in TBtools^[^ ^71^ ^]^ (e‐value = 1e‐5, NumofHits = 5). The full‐length transcripts were aligned in ClastalW and constructed the NJ tree with 1000 bootstrap steps in MEGA11.^[^ ^73^ ^]^ The expression level data of each unigenes were extracted from the TPM matrix and used for drawing heatmaps by Tbtools.^[^ ^71^ ^]^

Only one of the unigenes with highly similar CDS was retained for subsequent experiments. RNA levels of the remaining four TCP genes (i1_HQ_lanhua_c24148/f3p1/1714, i1_LQ_lanhua_c47139/f1p12/1518, i1_LQ_lanhua_c8646/f1p9/1748, and i1_LQ_lanhua_c20715/f1p0/1521) were analyzed by qRTPCR used another three individuals from different cloned line (LY1, LY2, and LY3). The seeds of these individuals were collected from wild populations in southwest China and cultivated at the greenhouse of Lvyuan Co. Ltd., Guizhou, China under a standardized condition. ACTIN (i1_LQ_lanhua_c44376/f7p0/1657) was selected as the reference gene for the normalization of the data. Primer sequences are listed in Table S15 (Supporting Information). The total RNA was extracted from root, stem, leaf, and flower bud (≈4 cm long) using the HiPure Plant RNA Plus Kit (catalog no. R4150, Magen, Guangzhou, China). The total RNA was reverse transcribed in a 20 µl volume using Random Primers N6 using the Hifair II 1st Strand cDNA Synthesis Kit (catalog no. 11119ES60, Yeasen, Shanghai, China). All samples were run in triplicate using the ABI ViiA 7 Real‐Time PCR System (Applied Biosystems, CA, USA). The amplification program was performed as follows: 5 min s at 95 °C for initial denaturation, 40 cycles of 10 s at 95 °C for denaturation, 30 s at 60 °C for annealing and elongation, followed by melting curve program for melting curve analysis. The expression level was calculated by the 2^−ΔCT^ method to compare across samples and genes.

Genotyping of the candidate eSSR in i1_HQ_lanhua_c24148/f3p1/1714 was conducted using CE. SSR primers were designed using Primer3 software^[^ ^74^ ^]^ as follows: forward primer 5’‐TCCCTCCTCTCCAGTCCATC‐3’ and reverse primer 5’‐ TCGTGAGACCAACTACAGGC‐3’. Total genomic DNA was extracted using the HiPure Plant DNA Kits (catalog no. D3163, Magen, Guangzhou, China). The forward primers of the SSR locus were labeled with fluorescence dye 6‐FAM. A 20 µL PCR reaction system was used, which includes primer F (1 µм) and primer R (1 µм), template DNA (2 µL), and 2x Taq Master Mix (10 µL). The PCR reaction procedure is 94 °C 10 min^−1^; 28 cycles of 94 °C 30 s^−1^, 58 °C 30 s^−1^, and 72 °C 20 s^−1^; 72 °C 5 min^−1^. The amplicons were sent for CE (Sengon Bio Co., Shanghai, China). PCR products were diluted 1:100 and run with internal standard LIZ‐500 on an ABI 3730 genetic analyzer (Applied Biosystems, Warrington, Cheshire, UK). Allelic sizes were scored using Gene Mapper 4.0 software (Applied Biosystems, Warrington, Cheshire, UK).

## Conflict of Interest

The authors declare no conflict of interest.

## Author Contributions

T.W., Z.J.L., Y.Y.L., and Y.J.S. conceived and designed the study. Z.J.L., G.Q.Z., and Y.Y.L. collected the *P. malipoense* materials and performed the RNA‐seq. J.Y.W. and Y.Y.L. prepared the samples for third‐generation sequencing. Y.Y.L., J.H., and J.Y.W. performed the experiments and analyzed the data. Y.Y.L., J.H., and T.W. interpreted the data and drafted the manuscript, and all authors contributed to the final version of the manuscript. Y.Y.L. and J.H. prepared the Figures.

## Supporting information

Supporting Information

Supporting Table 1

## Data Availability

The raw sequencing data generated in this study have been deposited at NCBI under accession number PRJNA968160. The data that support the findings of this study are available in the supplementary material of this article.

## References

[1] M. Gymrek , T. Willems , A. Guilmatre , H. Zeng , B. Markus , S. Georgiev , M. J. Daly , A. L. Price , J. K. Pritchard , A. J. Sharp , Y. Erlich , Nat. Genet 2016, 48, 22. 26642241 10.1038/ng.3461 PMC4909355

[2] M. Bakhtiari , J. Park , Y.‐C. Ding , S. Shleizer‐Burko , S. L. Neuhausen , B. V. Halldórsson , K. Stefánsson , M. Gymrek , V. Bafna , Nat. Commun. 2021, 12, 2075. 33824302 10.1038/s41467-021-22206-z PMC8024321

[3] G. S. Erwin , G. Gürsoy , R. Al‐Abri , A. Suriyaprakash , E. Dolzhenko , K. Zhu , C. R. Hoerner , S. M. White , L. Ramirez , A. Vadlakonda , A. Vadlakonda , K. Von Kraut , J. Park , C. M. Brannon , D. A. Sumano , R. A. Kirtikar , A. A. Erwin , T. J. Metzner , R. K. C. Yuen , A. C. Fan , J. T. Leppert , M. A. Eberle , M. Gerstein , M. P. Snyder , Nature 2023, 613, 96. 36517591 10.1038/s41586-022-05515-1 PMC9812771

[4] S. F. Fotsing , J. Margoliash , C. Wang , S. Saini , R. Yanicky , S. Shleizer‐Burko , A. Goren , M. Gymrek , Nat. Genet 2019, 51, 1652. 31676866 10.1038/s41588-019-0521-9 PMC6917484

[5] M. Verbiest , M. Maksimov , Y. Jin , M. Anisimova , M. Gymrek , T. Bilgin Sonay , J. Evolution. Biol. 2023, 36, 321. 10.1111/jeb.14106 PMC9990875 36289560

[6] C. Gao , X. Ren , A. S. Mason , J. Li , W. Wang , M. Xiao , D. Fu , Funct. Plant Biol. 2013, 40, 645. 32481138 10.1071/FP12325

[7] S. Sureshkumar , M. Todesco , K. Schneeberger , R. Harilal , S. Balasubramanian , D. Weigel , Science 2009, 323, 1060. 19150812 10.1126/science.1164014

[8] S. Kumar , S. Bhatia , Sci. Rep. 2016, 6, 33280. 27623355 10.1038/srep33280 PMC5020687

[9] V. Y. Vavilova , I. D. Konopatskaia , A. G. Blinov , E. Y. Kondratenko , Y. V. Kruchinina , N. P. Goncharov , Russ. J. Genet 2022, 58, 684.

[10] C. Zhang , C. Yu , T. Zhang , W. Guo , X. Tian , J. Integr. Agric. 2021, 20, 2180.

[11] P. Rival , M. O. Press , J. Bale , T. Grancharova , S. F. Undurraga , C. Queitsch , Genetics 2014, 198, 747. 25116137 10.1534/genetics.114.167866 PMC4196625

[12] M. O. Press , C. Queitsch , Genetics 2017, 205, 455. 27866166 10.1534/genetics.116.193359 PMC5223521

[13] Y.‐C. Li , Mol. Biol. Evol. 2004, 21, 991. 14963101 10.1093/molbev/msh073

[14] V. Dwivedi , L. Pal , D. Yadav , in Bioinformatics in Agriculture, Elsevier, Amsterdam, Netherlands 2022, pp. 537.

[15] C. Ranathunge , G. L. Wheeler , M. E. Chimahusky , M. M. Kennedy , J. I. Morrison , B. S. Baldwin , A. D. Perkins , M. E. Welch , Mol. Ecol. 2018, 27, 1188. 29419922 10.1111/mec.14522

[16] C. Ranathunge , G. L. Wheeler , M. E. Chimahusky , A. D. Perkins , S. Pramod , M. E. Welch , Mol. Ecol. 2020, 29, 1704. 32285554 10.1111/mec.15440

[17] C. Ranathunge , S. Pramod , S. Renaut , G. L. Wheeler , A. D. Perkins , L. H. Rieseberg , M. E. Welch , Symmetry 2021, 13, 933.

[18] L. Xu , R. J. Haasl , J. Sun , Y. Zhou , D. M. Bickhart , J. Li , J. Song , T. S. Sonstegard , C. P. Van Tassell , H. A. Lewin , G. E. Liu , Genome Biol. Evol. 2016, 9, 20. 10.1093/gbe/evw256 PMC5381564 28172841

[19] M. O. Press , R. C. McCoy , A. N. Hall , J. M. Akey , C. Queitsch , Genome Res. 2018, 28, 1169. 29970452 10.1101/gr.231753.117 PMC6071631

[20] C. Ranathunge , M. E. Chimahusky , M. E. Welch , Mol. Genet Genomics 2022, 297, 1329. 35786764 10.1007/s00438-022-01920-3

[21] M. D. Vinces , M. Legendre , M. Caldara , M. Hagihara , K. J. Verstrepen , Science 2009, 324, 1213. 19478187 10.1126/science.1170097 PMC3132887

[22] G.‐Q. Zhang , K.‐W. Liu , Z. Li , R. Lohaus , Y.‐Y. Hsiao , S.‐C. Niu , J.‐Y. Wang , Y.‐C. Lin , Q. Xu , L.‐J. Chen , K. Yoshida , S. Fujiwara , Z.‐W. Wang , Y.‐Q. Zhang , N. Mitsuda , M. Wang , G.‐H. Liu , L. Pecoraro , H.‐X. Huang , X.‐J. Xiao , M. Lin , X.‐Y. Wu , W.‐L. Wu , Y.‐Y. Chen , S.‐B. Chang , S. Sakamoto , M. Ohme‐Takagi , M. Yagi , S.‐J. Zeng , C.‐Y. Shen , Nature 2017, 549, 379. 28902843 10.1038/nature23897 PMC7416622

[23] M.‐H. Li , K.‐W. Liu , Z. Li , H.‐C. Lu , Q.‐L. Ye , D. Zhang , J.‐Y. Wang , Y.‐F. Li , Z.‐M. Zhong , X. Liu , X. Yu , D.‐K. Liu , X.‐D. Tu , B. Liu , Y. Hao , X.‐Y. Liao , Y.‐T. Jiang , W.‐H. Sun , J. Chen , Y.‐Q. Chen , Y. Ai , J.‐W. Zhai , S.‐S. Wu , Z. Zhou , Y.‐Y. Hsiao , W.‐L. Wu , Y.‐Y. Chen , Y.‐F. Lin , J.‐L. Hsu , C.‐Y. Li , Nat. Plants 2022, 8, 373. 35449401 10.1038/s41477-022-01127-9 PMC9023349

[24] J. Pellicer , I. J. Leitch , New Phytol. 2020, 226, 301. 31608445 10.1111/nph.16261

[25] S. A. Unruh , M. R. McKain , Y. Lee , T. Yukawa , M. K. McCormick , R. P. Shefferson , A. Smithson , J. H. Leebens‐Mack , J. C. Pires , Am J. Bot. 2018, 105, 631. 29608785 10.1002/ajb2.1047

[26] J. W. Yap , Molecular and Genome Evolution in the Malesian Slipper Orchids (Paphiopedilum Section Barbata)., PhD, Queen Mary University of London, London 2016.

[27] T. Sun , F. Zhou , X.‐Q. Huang , W.‐C. Chen , M.‐J. Kong , C.‐F. Zhou , Z. Zhuang , L. Li , S. Lu , Plant Cell 2019, 31, 2996. 31604812 10.1105/tpc.18.00290 PMC6925005

[28] M. Kieffer , V. Master , R. Waites , B. Davies , Plant J. 2011, 68, 147. 21668538 10.1111/j.1365-313X.2011.04674.x PMC3229714

[29] E. Steiner , I. Efroni , M. Gopalraj , K. Saathoff , T.‐S. Tseng , M. Kieffer , Y. Eshed , N. Olszewski , D. Weiss , Plant Cell 2012, 24, 96. 22267487 10.1105/tpc.111.093518 PMC3289577

[30] L. Chen , H. Dong , J. Wang , L. Li , M. Xu , Plant Genet Resour. 2018, 16, 394.

[31] C.‐C. Tsai , H.‐C. Shih , H.‐V. Wang , Y.‐S. Lin , C.‐H. Chang , Y.‐C. Chiang , C.‐H. Chou , PLoS One 2015, 10, e0141761. 26523377 10.1371/journal.pone.0141761 PMC4629892

[32] X. Guo , Y. Li , C. Li , H. Luo , L. Wang , J. Qian , X. Luo , L. Xiang , J. Song , C. Sun , H. Xu , H. Yao , S. Chen , Gene 2013, 527, 131. 23756193 10.1016/j.gene.2013.05.073

[33] X. Li , J. Luo , T. Yan , L. Xiang , F. Jin , D. Qin , C. Sun , M. Xie , PLoS One 2013, 8, e85480. 24392013 10.1371/journal.pone.0085480 PMC3877369

[34] D. Xu , H. Chen , M. Aci , Y. Pan , Y. Shangguan , J. Ma , L. Li , G. Qian , Q. Wang , PLoS One 2018, 13, e0205954. 30365506 10.1371/journal.pone.0205954 PMC6203367

[35] B. A. Payseur , P. Jing , R. J. Haasl , Mol. Biol. Evol. 2011, 28, 303. 20675409 10.1093/molbev/msq198 PMC3002246

[36] R. Xu , Z. Wang , Y. Su , T. Wang , Front Genet 2020, 11, 574304. 33193679 10.3389/fgene.2020.574304 PMC7593448

[37] Y.‐H. Wang , Front Biosci. 2007, 12, 4731. 17485409 10.2741/2422

[38] F. Mignone , C. Gissi , S. Liuni , G. Pesole , Genome Biol. 2002, 3, reviews0004. 11897027 10.1186/gb-2002-3-3-reviews0004 PMC139023

[39] A. F. Castro , J. R. Loureiro , J. Bessa , I. Silveira , Genes 2020, 11, 1418. 33261024 10.3390/genes11121418 PMC7760973

[40] P. Calabrese , R. Sainudiin , in Statistical Methods in Molecular Evolution (Ed.: R. Nielsen ), Springer, New York, NY, 2005, pp. 290.

[41] The BRIDGES Consortium , J. Carlson , A. E. Locke , M. Flickinger , M. Zawistowski , S. Levy , R. M. Myers , M. Boehnke , H. M. Kang , L. J. Scott , J. Z. Li , S. Zöllner , D. Absher , H. Akil , G. Breen , M. Burmeister , S. Cohen‐Woods , W. G. Iacono , J. A. Knowles , L. Legrand , Q. Lu , M. McGue , M. G. McInnis , C. N. Pato , M. T. Pato , M. Rivera , J. L. Sobell , J. B. Vincent , S. J. Watson , Nat. Commun. 2018, 9, 3753. 30218074 10.1038/s41467-018-05936-5 PMC6138700

[42] A. Couce , O. Tenaillon , Nat. Commun. 2019, 10, 3114. 31308380 10.1038/s41467-019-11217-6 PMC6629674

[43] L. Zhang , D. Yuan , S. Yu , Z. Li , Y. Cao , Z. Miao , H. Qian , K. Tang , Bioinformatics 2004, 20, 1081. 14764542 10.1093/bioinformatics/bth043

[44] R. A. Martienssen , V. Colot , Science 2001, 293, 1070. 11498574 10.1126/science.293.5532.1070

[45] I. Sangwan , M. R. O'Brian , Plant Physiol. 2002, 129, 1788. 12177492 10.1104/pp.002618 PMC166767

[46] N. Abdul‐Manan , Nucleic Acids Res. 1996, 24, 4063. 8918813 10.1093/nar/24.20.4063 PMC146211

[47] M. J. Matunis , J. Xing , G. Dreyfuss , Nucleic Acids Res. 1994, 22, 1059. 7512260 10.1093/nar/22.6.1059 PMC307930

[48] P. S. Bagga , G. K. Arhin , J. Wilusz , Nucleic Acids Res. 1998, 26, 5343. 9826757 10.1093/nar/26.23.5343 PMC147992

[49] Y. Hew , C. Lau , Z. Grzelczak , F. W. Keeley , J. Biol. Chem 2000, 275, 24857. 10829024 10.1074/jbc.M002776200

[50] J. Hao , Y. Liang , J. Ping , J. Li , W. Shi , Y. Su , T. Wang , BMC Plant Biol. 2022, 22, 580. 36510137 10.1186/s12870-022-03960-8 PMC9746204

[51] A. J. Gruber , M. Zavolan , Nat. Rev. Genet 2019, 20, 599. 31267064 10.1038/s41576-019-0145-z

[52] K. Kikuchi , Y. Sato , E. C. M. Juan , A. Takénaka , Nucleic Acids Symp. Ser. 2006, 50, 217. 10.1093/nass/nrl108 17150895

[53] T. L. Hotchkiss , G. E. Nerantzakis , S. C. Dills , L. Shang , L. K. Read , Mol. Biochem. Parasitol. 1999, 98, 117. 10029314 10.1016/s0166-6851(98)00156-x

[54] Q. Yang , B. Grimmig , U. Matern , Plant Mol. Biol. 1998, 38, 1201. 9869425 10.1023/a:1006003731919

[55] J. L. Wilding , S. McGowan , Y. Liu , W. F. Bodmer , Proc. Natl. Acad. Sci., USA 2010, 107, 21058. 21097699 10.1073/pnas.1015604107 PMC3000307

[56] S. Kishore , S. Piscuoglio , M. B. Kovac , A. Gylling , F. Wenzel , F. Trapani , H. J. Altermatt , V. Mele , G. Marra , P. Peltomäki , L. Terracciano , M. Zavolan , K. Heinimann , Cancer Res. 2014, 74, 224. 24158095 10.1158/0008-5472.CAN-13-2100

[57] J. Shin , Z. Yuan , K. Fordyce , P. Sreeramoju , T. S. Kent , J. Kim , V. Wang , D. Schneyer , T. K. Weber , Surgery 2007, 142, 222. 17689689 10.1016/j.surg.2007.04.002

[58] T. Ruggiero , Nucleic Acids Res. 2003, 31, 6561. 14602915 10.1093/nar/gkg858 PMC275554

[59] N. V. Hoang , A. Furtado , P. J. Mason , A. Marquardt , L. Kasirajan , P. P. Thirugnanasambandam , F. C. Botha , R. J. Henry , BMC Genomics 2017, 18, 395. 28532419 10.1186/s12864-017-3757-8 PMC5440902

[60] W. Li , A. Godzik , Bioinformatics 2006, 22, 1658. 16731699 10.1093/bioinformatics/btl158

[61] C. Camacho , G. Coulouris , V. Avagyan , N. Ma , J. Papadopoulos , K. Bealer , T. L. Madden , BMC Bioinformatics 2009, 10, 421. 20003500 10.1186/1471-2105-10-421 PMC2803857

[62] C. P. Cantalapiedra , A. Hernández‐Plaza , I. Letunic , P. Bork , J. Huerta‐Cepas , Mol. Biol. Evol. 2021, 38, 5825. 34597405 10.1093/molbev/msab293 PMC8662613

[63] Y. Moriya , M. Itoh , S. Okuda , A. C. Yoshizawa , M. Kanehisa , Nucleic Acids Res. 2007, 35, W182. 17526522 10.1093/nar/gkm321 PMC1933193

[64] E. L. L. Sonnhammer , S. R. Eddy , R. Durbin , Proteins 1997, 28, 405. 9223186 10.1002/(sici)1097-0134(199707)28:3<405::aid-prot10>3.0.co;2-l

[65] Y.‐J. Kang , D.‐C. Yang , L. Kong , M. Hou , Y.‐Q. Meng , L. Wei , G. Gao , Nucleic Acids Res. 2017, 45, W12. 28521017 10.1093/nar/gkx428 PMC5793834

[66] L. Sun , H. Luo , D. Bu , G. Zhao , K. Yu , C. Zhang , Y. Liu , R. Chen , Y. Zhao , Nucleic Acids Res. 2013, 41, e166. 23892401 10.1093/nar/gkt646 PMC3783192

[67] L. Wang , H. J. Park , S. Dasari , S. Wang , J.‐P. Kocher , W. Li , Nucleic Acids Res. 2013, 41, e74. 23335781 10.1093/nar/gkt006 PMC3616698

[68] A. Li , J. Zhang , Z. Zhou , BMC Bioinformatics 2014, 15, 311. 25239089 10.1186/1471-2105-15-311 PMC4177586

[69] W. H. Kruskal , W. A. Wallis , J. Am. Stat. Assoc. 1952, 47, 583.

[70] H. Wickham , Ggplot2: Elegant Graphics for Data Analysis, Springer, New York, 2009.

[71] C. Chen , H. Chen , Y. Zhang , H. R. Thomas , M. H. Frank , Y. He , R. Xia , Mol. Plant 2020, 13, 1194. 32585190 10.1016/j.molp.2020.06.009

[72] Z. Gu , D. Hübschmann , Genom. Proteom. Bioinf. 2023, 21, 190. 10.1016/j.gpb.2022.04.008 PMC10373083 35680096

[73] K. Tamura , G. Stecher , S. Kumar , Mol. Biol. Evol. 2021, 38, 3022. 33892491 10.1093/molbev/msab120 PMC8233496

[74] A. Untergasser , I. Cutcutache , T. Koressaar , J. Ye , B. C. Faircloth , M. Remm , S. G. Rozen , Nucleic Acids Res. 2012, 40, e115. 22730293 10.1093/nar/gks596 PMC3424584

