# Supplementary material for: Statistical Genomics Analysis of Simple Sequence Repeats from the Paphiopedilum Malipoense Transcriptome Reveals Control Knob Motifs Modulating Gene Expression
Source: Adv Sci (Weinh). 2024 Apr 22;11(24):2304848. doi: 10.1002/advs.202304848 (PMC11200097; doi:10.1002/advs.202304848)
Supplement: Supplementary file 1 — Supporting Information [file ADVS-11-2304848-s002.pdf]

ADVANCED SCIENCE
Open Access

Supporting Information

for *Adv. Sci.*, DOI 10.1002/advs.202304848

Statistical Genomics Analysis of Simple Sequence Repeats from the *Paphiopedilum Malipoense* Transcriptome Reveals Control Knob Motifs Modulating Gene Expression

Yingyi Liang, Jing Hao, Jieyu Wang, Guoqiang Zhang, Yingjuan Su, Zhong-Jian Liu* and Ting Wang**

Supporting Information

Statistical genomics analysis of simple sequence repeats from the *Paphiopedilum malipoense* transcriptome reveals control knob motifs that modulate gene expression.

Yingyi Liang, Jing Hao, Jieyu Wang, Guoqiang Zhang, Yingjuan Su, Zhong-Jian Liu*, Ting Wang**

Content

Additional file 1: Figures **4**

Figure S1. Length of six SSR repeat types in the *P. malipoense* transcriptome.... 4

Figure S2. Length of SSRs in the three transcribed regions. 5

Figure S3. SSR length of different motif sizes in the three transcribed regions.... 6

Figure S4. Adjusted standardized residual from chi-square test on frequencies between standardized mononucleotides in the *P. malipoense* transcriptome........ 7

Figure S5. Adjusted standardized residual from the chi-square test on frequencies between standardized dinucleotides in *the P. malipoense* transcriptome........ 8

Figure S6. Adjusted standardized residual from chi-square test on frequencies between standardized trinucleotides in the *P. malipoense* transcriptome........ 9

Figure S7. Comparisons of SSR characteristics among SSR-containing transcripts with different expression signatures........ 10

Figure S8. Distribution patterns of different motif size SSRs at various expression levels. 12

Figure S9. Distribution patterns of SSRs in lncRNAs at various expression levels. 13

Figure S10. Comparisons of SSR characteristics among SSR-containing lncRNAs with different expression signatures. 15

Figure S11. Regression models based on $lnTPM_{max}$ and SSR density of fully standardized expMotifs within the 5’-UTR. 17

Figure S12. Regression models based on $lnTPM_{max}$ and SSR density of fully standardized expMotifs within CDS. 19

Figure S13. Regression models based on $lnTPM_{max}$ and SSR density of fully standardized expMotifs within the 3’-UTR.. 20

Figure S14. Regression models based on $lnTPM_{CV}$ and SSR density of fully standardized expMotifs within three transcribed regions. 21

Figure S15. Regression models based on $lnTPM_{max}$ and SSR density of actual expMotifs within the 5’-UTR and CDS........ 22

Figure S16. Regression models based on $lnTPM_{max}$ and SSR density of actual expMotifs within the 3’-UTR. 24

Figure S17. Regression models based on $lnTPM_{CV}$ and SSR characteristics of actual expMotifs within transcribed regions. 26
Figure S18. Profiles of expMotif-SSRs at TCP gene i1_HQ_lanhua_c24148/f3p1/1714 in three individuals by CE. 28
Additional file 1: Tables 30
Table S1. Statistics of PacBio sequencing of the *P. malipoense* transcriptome. 30
Table S2. Summary of the functional annotation of the reference transcriptome of *P. malipoense*. 31
Table S3. Kruskal–Wallis test results of TPM_{max} among transcribed regions with different motif sizes of SSRs. 31
Table S4. Kruskal–Wallis test results of TPM_{CV} among transcribed regions with different motif sizes of SSRs. 32
Table S5. Fully standardized motifs distributed in different transcribed regions had significantly different TPM_{max} values. 32
Table S6. Fully standardized motifs distributed in different transcribed regions had significantly different TPM_{CV} values. 33
Table S7. The optimal models of fully standardized motif characteristics and $lnTPM_{max}$. 34
Table S8. The optimal models of fully standardized motif characteristics and $lnTPM_{CV}$. 35
Table S9. Summary of the results of statistical tests and regression analyses of 29 candidate expMotifs. 36
Table S10. Actual motifs distributed in different transcribed regions had significantly different TPM_{max} values. 39
Table S11. Actual motifs distributed in different transcribed regions had significantly different TPM_{CV} values. 40
Table S12. The optimal models of actual motif characteristics and $lnTPM_{max}$. 41
Table S13. The optimal models of actual motif characteristics and $lnTPM_{CV}$. 42
Table S14. Groping criteria for unigenes based on TPM_{max} and TPM_{CV} values. 43
Table S15. Primers used for qRTPCR. 43

Additional file 1: Figures

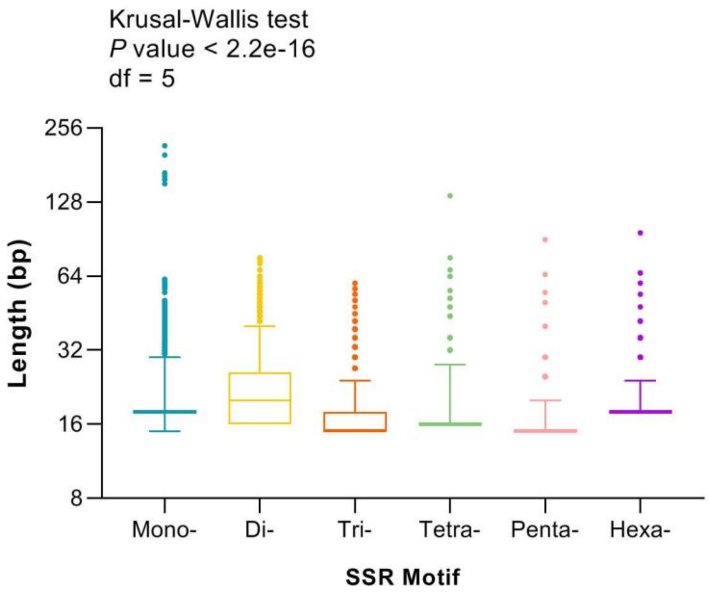

Figure S1. Length of six SSR repeat types in the *P. malipoense* transcriptome.

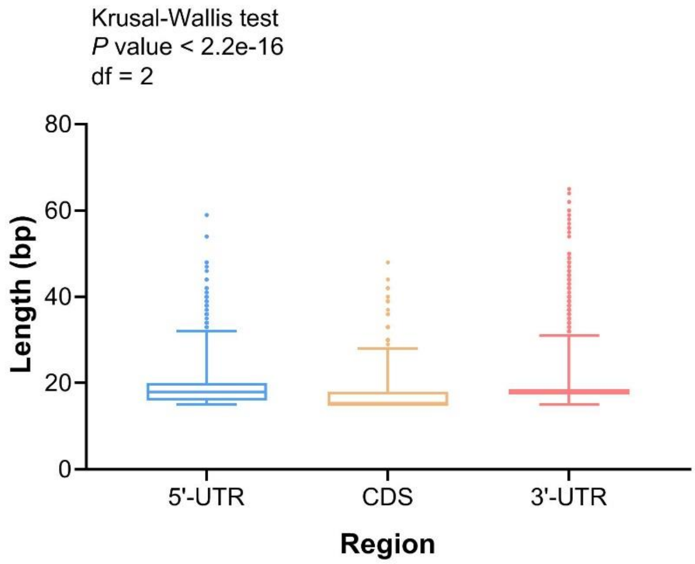

Figure S2. Length of SSRs in the three transcribed regions.

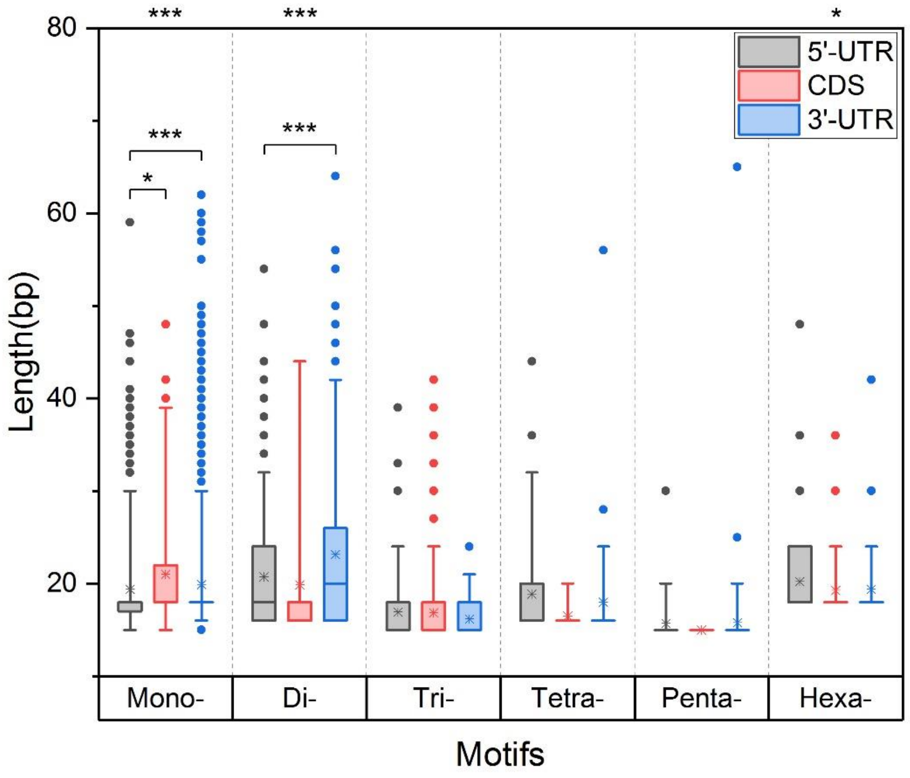

Figure S3. SSR length of different motif sizes in the three transcribed regions. * indicates a significant difference at $p < 0.05$, *** indicates a significant difference at $p < 0.001$ by the Kruskal–Wallis test and Dunn's pairwise test.

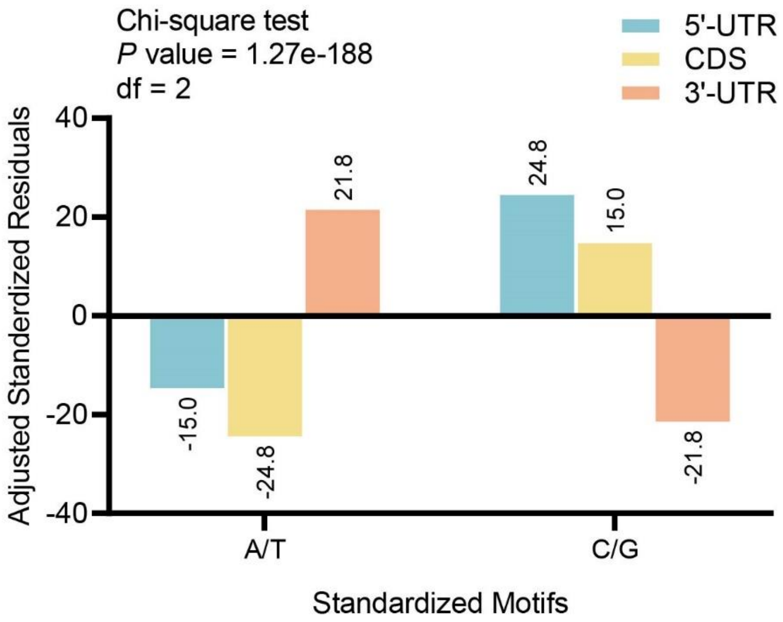

Figure S4. Adjusted standardized residual from chi-square test on frequencies between standardized mononucleotides in the *P. malipoense* transcriptome. This figure shows the deviation from the expectation of counts assuming no biased distribution of motifs among transcribed regions in the transcriptome.

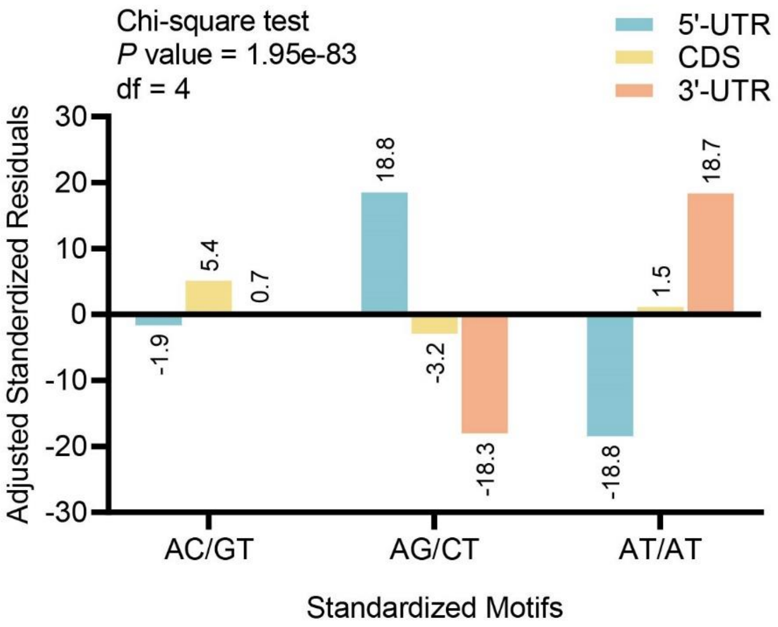

Figure S5. Adjusted standardized residual from the chi-square test on frequencies between standardized dinucleotides in *the P. malipoense* transcriptome. This figure shows the deviation from the expectation of counts assuming no biased distribution of motifs among transcribed regions in the transcriptome.

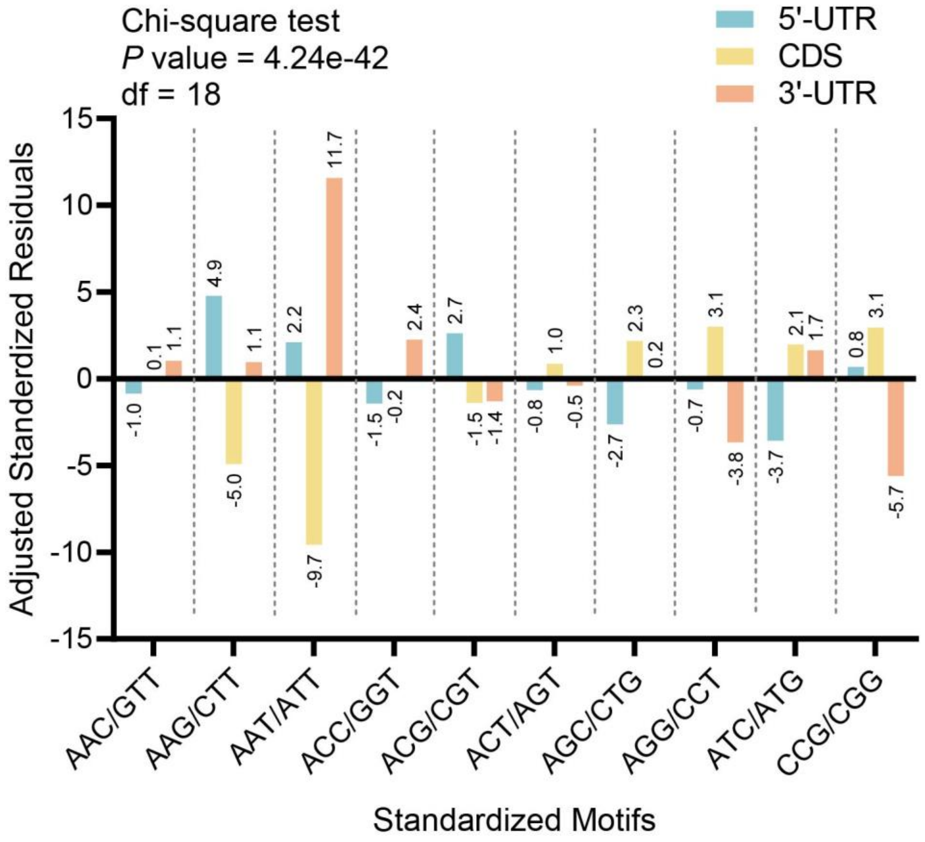

Figure S6. Adjusted standardized residual from chi-square test on frequencies between standardized trinucleotides in the *P. malipoense* transcriptome. This figure shows the deviation from the expectation of counts assuming no biased distribution of motifs among transcribed regions in the transcriptome.

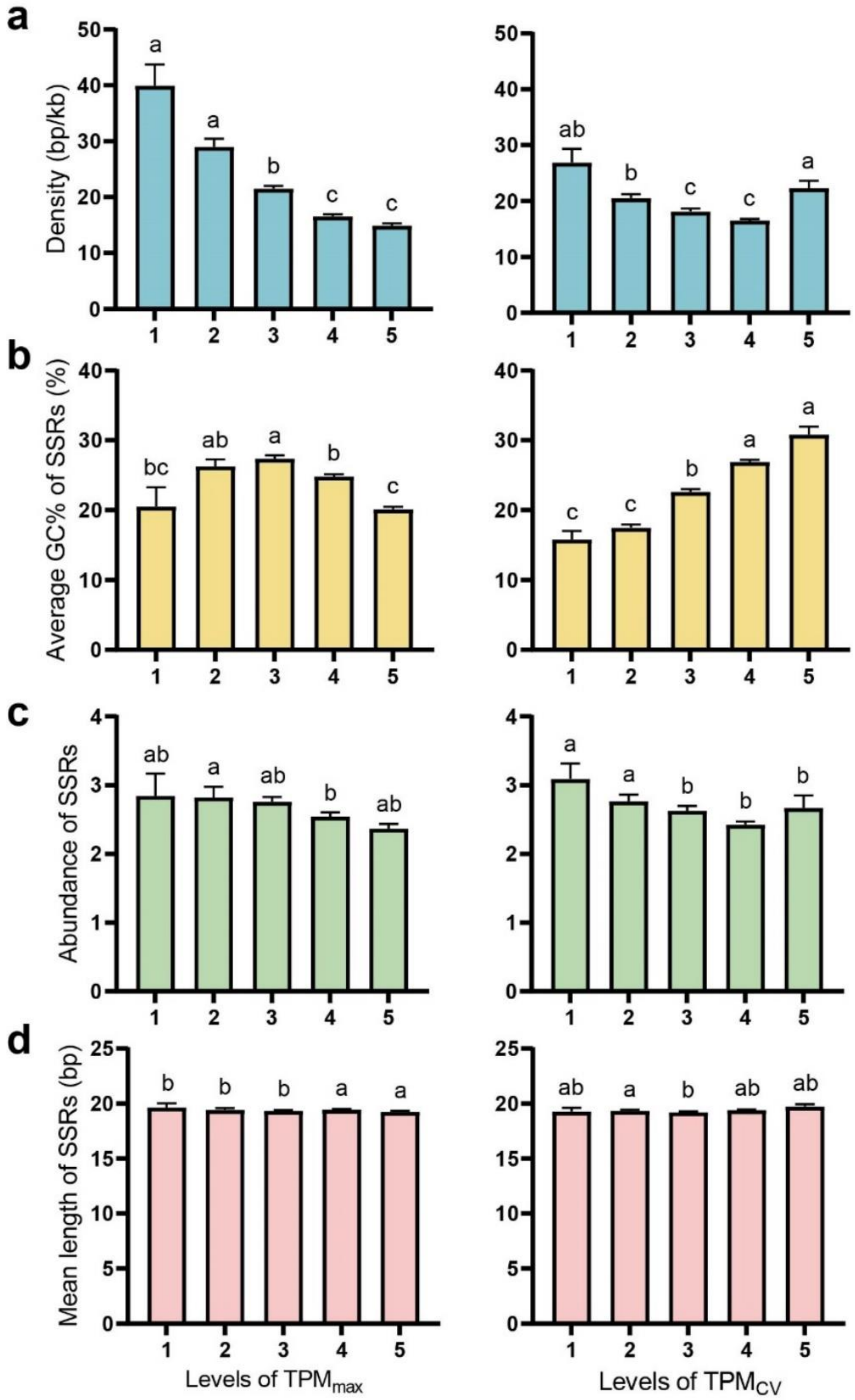

Figure S7. Comparisons of SSR characteristics among SSR-containing transcripts with different expression signatures. **(a)** The average SSR density among TPM_{max} (left) and TPM_{CV} (right) levels. **(b)** The average GC content of SSRs (within SSR-containing transcripts) among TPM_{max} (left) and TPM_{CV} (right) levels. **(c)** The average SSR abundance (within SSR-containing unigenes) among TPM_{max} (left) and TPM_{CV} (right) levels. **(d)** The mean length of SSRs among TPM_{max} (left) and TPM_{CV}

(right) levels. The error bar indicates the standard error of the mean, different letters represent significant differences, and the same letters represent no significant difference (Dunn's post hoc, p value < 0.05).

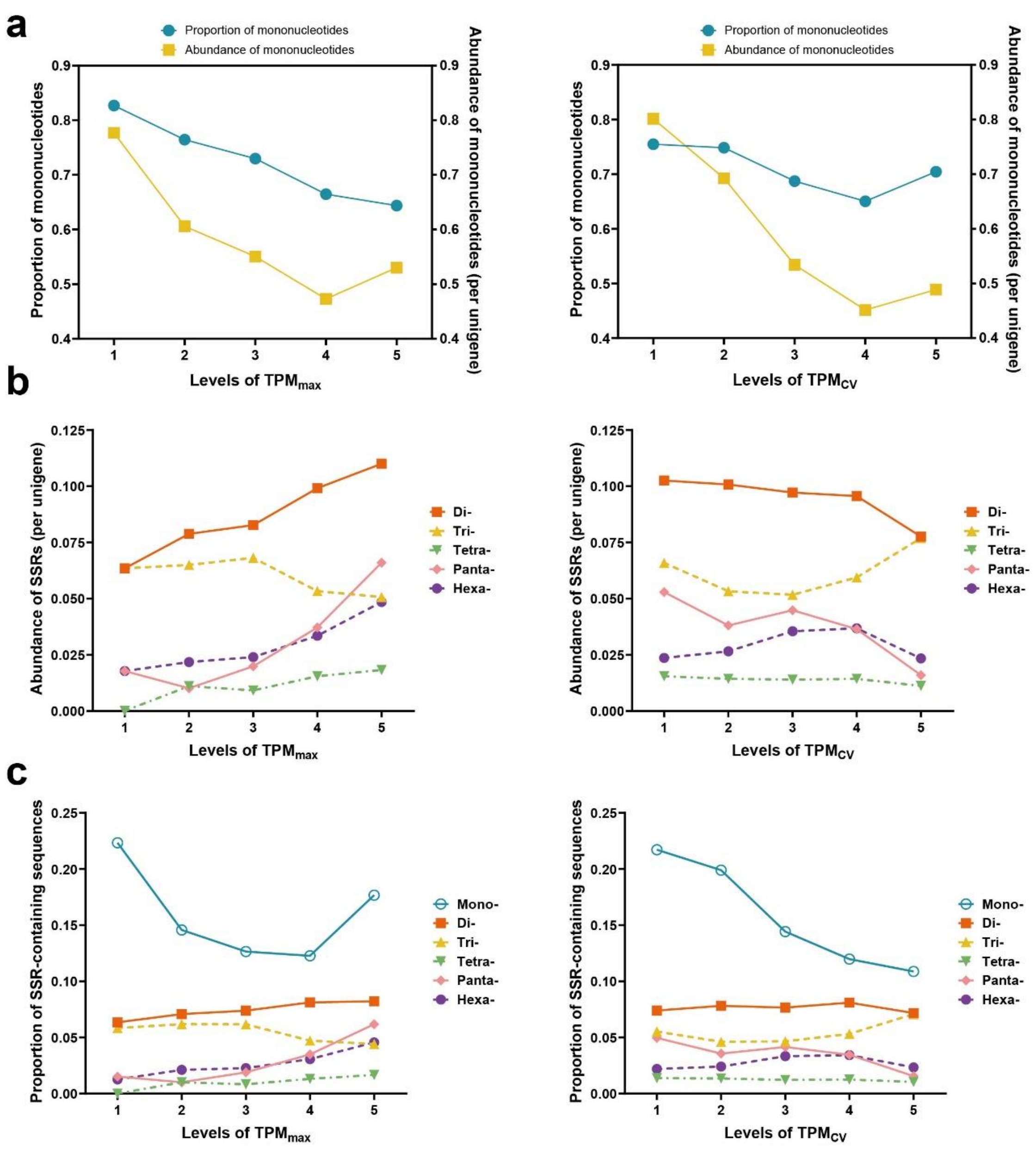

Figure S8. Distribution patterns of different motif size SSRs at various expression levels. **(a)** Proportion and abundance of mononucleotides, grouped by TPM_{max} (left) and TPM_{CV} (right). **(b)** Abundance of di-, tri-, tetra-, penta-, and hexa-nucleotides, grouped by TPM_{max} (left) and TPM_{CV} (right). **(c)** The proportion of sequences containing different motif size SSRs, grouped by TPM_{max} (left) and TPM_{CV} (right).

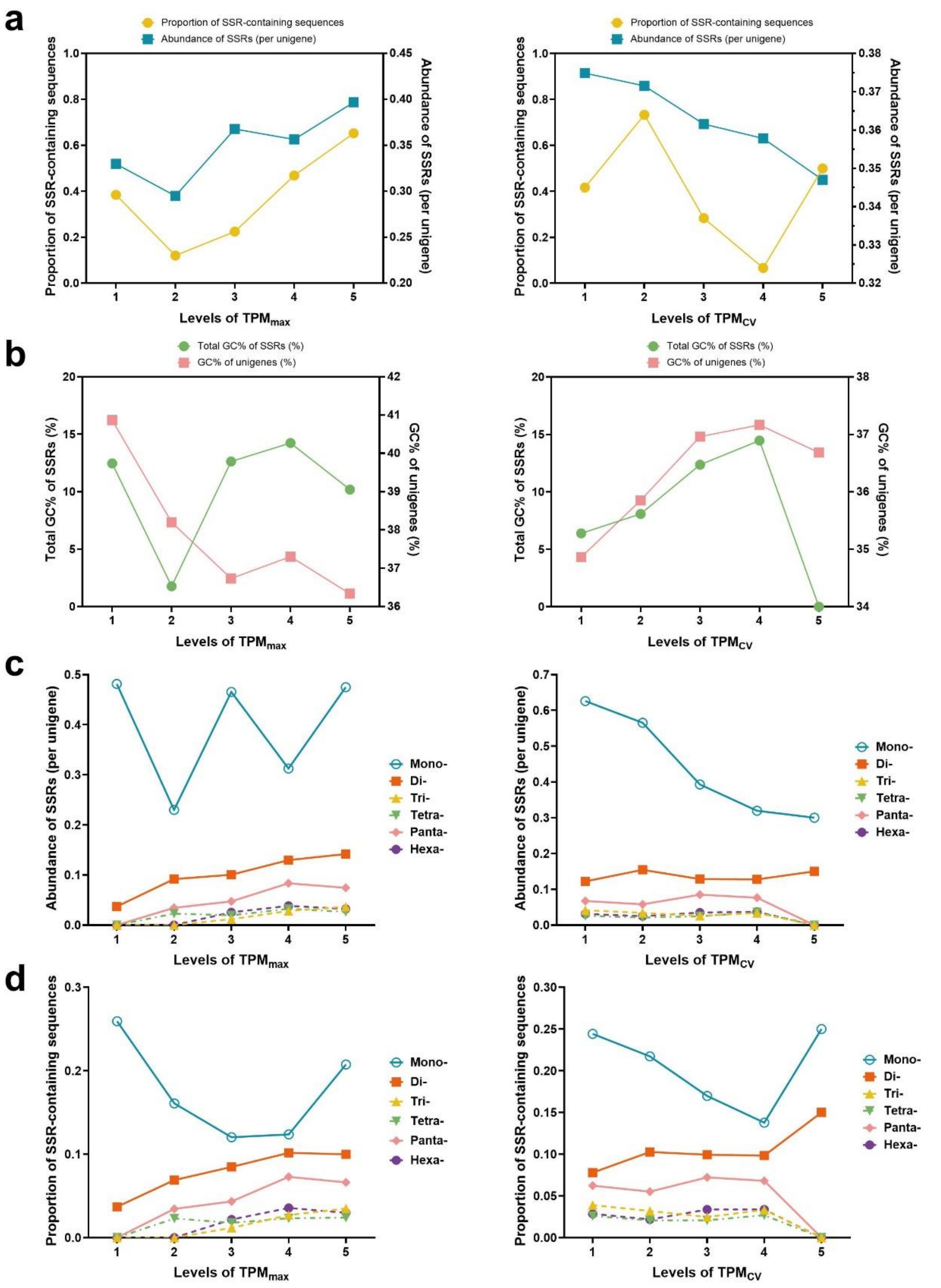

Figure S9. Distribution patterns of SSRs in lncRNAs at various expression levels. **(a)** Trends of the proportion of SSR-containing sequences and SSR abundance of lncRNAs as TPM_{max} (left) and TPM_{CV} (right) decreased. **(b)** Trends of the GC contents of SSRs in lncRNAs and their sequence contexts as TPM_{max} (left) and TPM_{CV} (right) decrease. **(c)** The abundance of mono-, di-, tri-, tetra-, penta-, and

hexa-nucleotides in lncRNAs, grouped by TPM_{max} (left) and TPM_{CV} (right). **(d)** The proportion of sequences containing different motif size SSRs in lncRNAs, grouped by TPM_{max} (left) and TPM_{CV} (right).

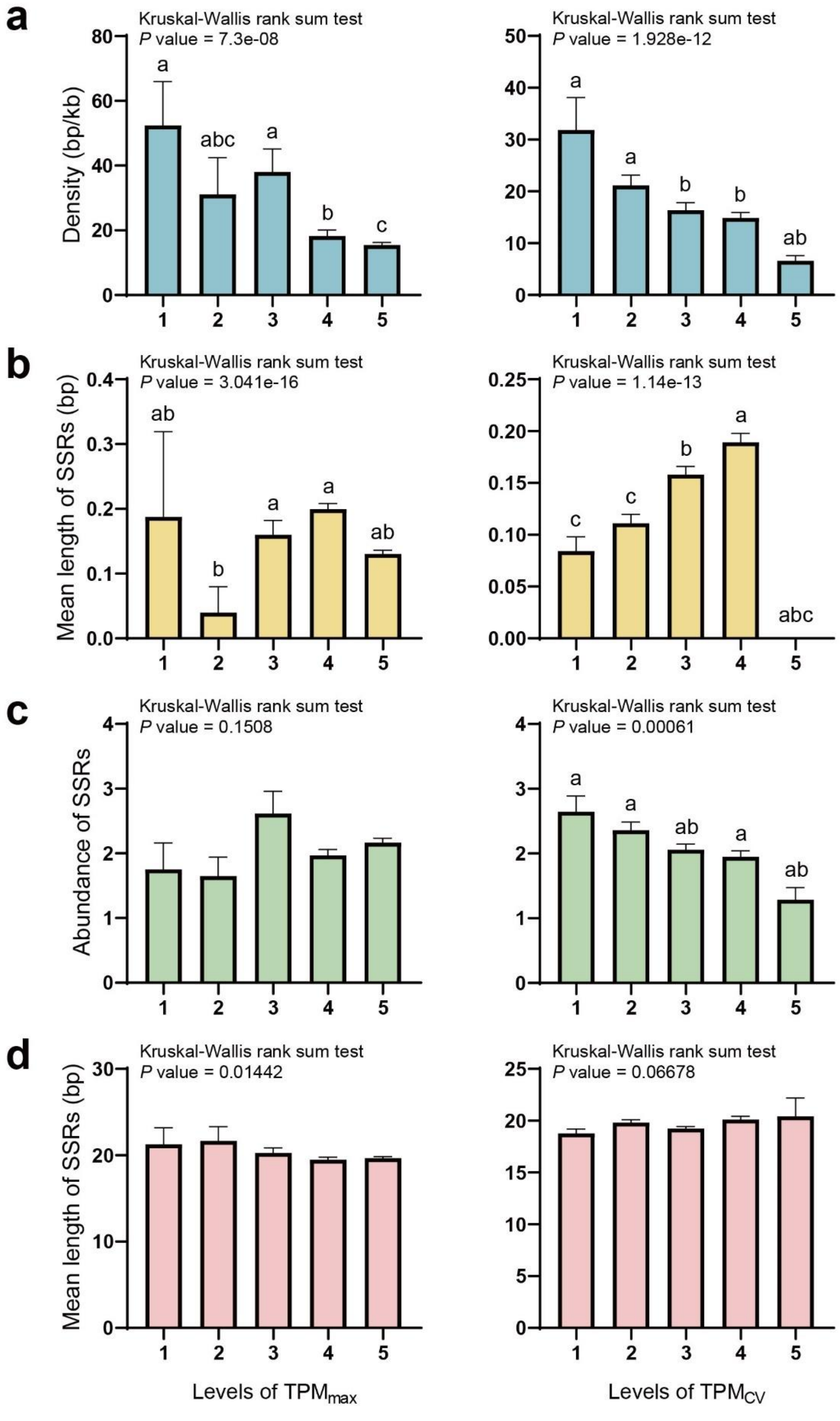

Figure S10. Comparisons of SSR characteristics among SSR-containing lncRNAs with different expression signatures. **(a)** The average SSR density among TPM_{max} (left) and TPM_{CV} (right) levels. **(b)** The average GC content of SSRs (within SSR-

containing transcripts) among TPM_{max} (left) and TPM_{CV} (right) levels. **(c)** The average SSR abundance (within SSR-containing unigenes) among TPM_{max} (left) and TPM_{CV} (right) levels. **(d)** Mean length of SSRs among TPM_{max} (left) and TPM_{CV} (right) levels. The error bar indicates the standard error of the mean, different letters represent significant differences, and the same letters represent no significant difference (Dunn's post hoc, p value < 0.05).

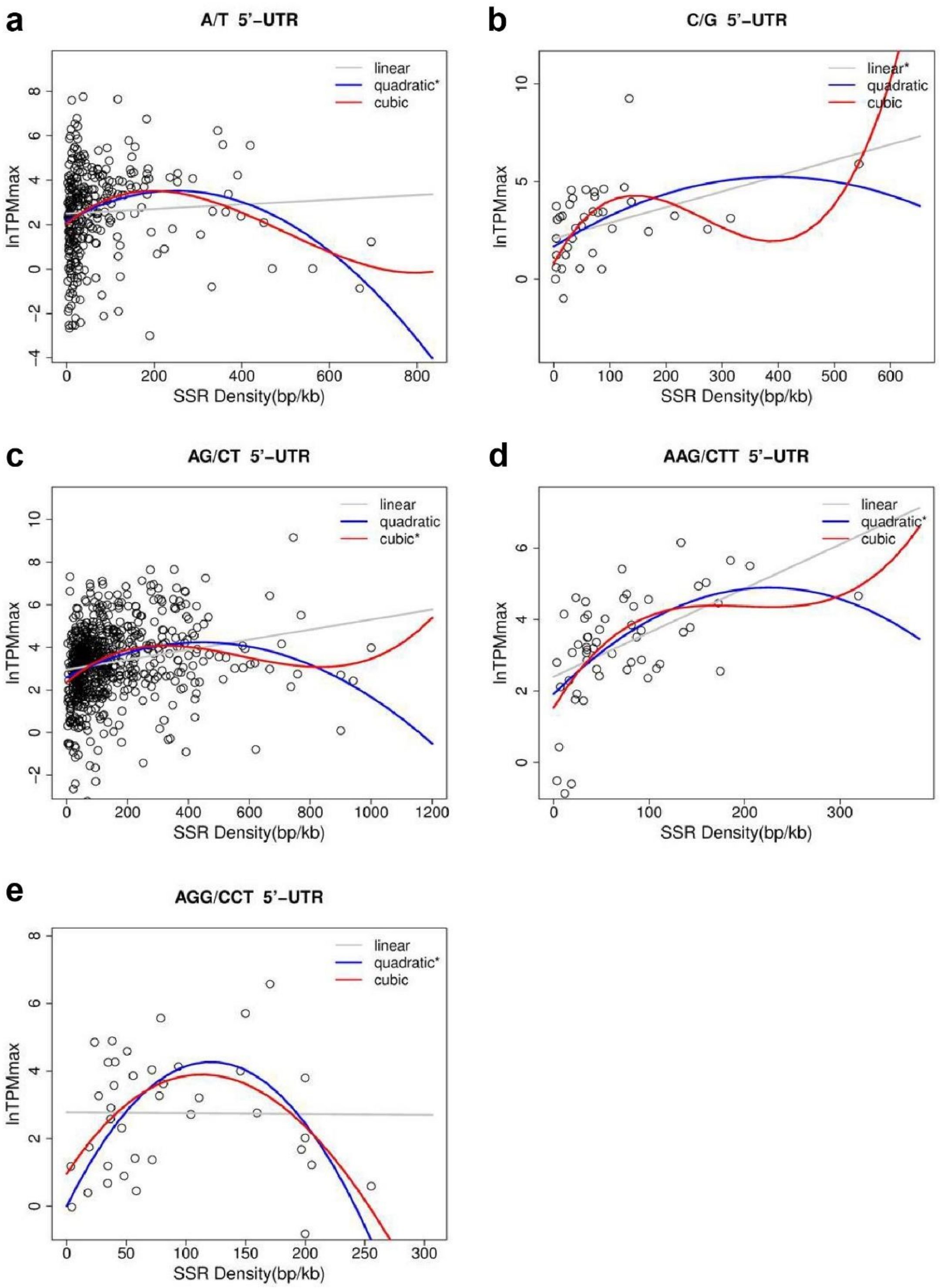

Figure S11. Regression models based on $lnTPM_{max}$ and SSR density of fully standardized expMotifs within the 5’-UTR. **(a)** Regression model for the correlation between the density of A/T repeats in 5’-UTR and $lnTPM_{max}$. **(b)** Regression model for the correlation between the density of C/G repeats in 5’-UTR and $lnTPM_{\mathbf{max}}$. **(c)** Regression model for the correlation between the density of AG/CT repeats in 5’-

UTR and $\ln TPM_{max}$. **(d)** Regression models for the correlation between the density of AAG/CTT repeats in 5’-UTR and $\ln TPM_{max}$. **(e)** Regression model for the correlation between the density of AGG/CCT repeats in 5’-UTR and $\ln TPM_{max}$. The asterisks indicate the optimal model ($p < 0.05$). The overfitting models and expMotif-region combinations with a sample size of less than 15 were excluded.

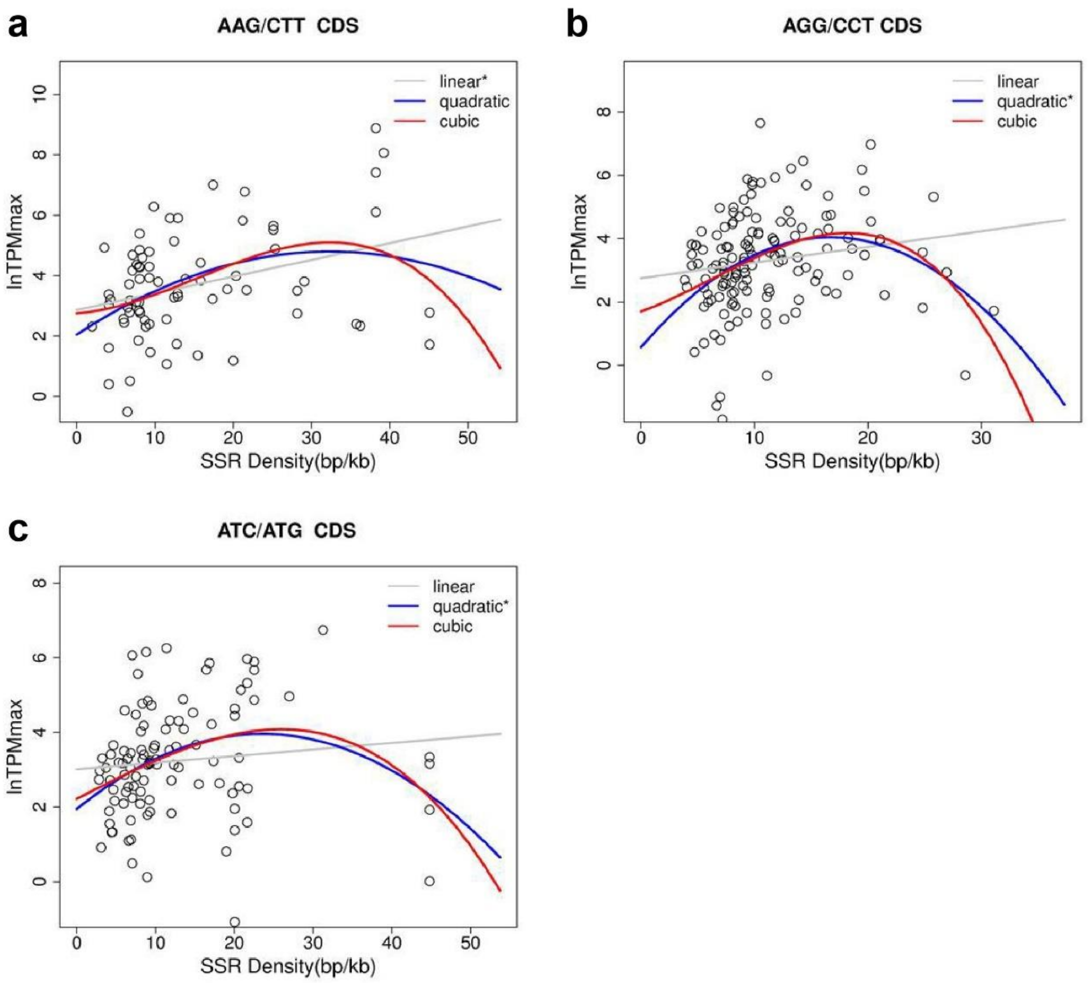

Figure S12. Regression models based on lnTPM$_{max}$ and SSR density of fully standardized expMotifs within CDS. **(a)** Regression model for the correlation between the density of AAG/CTT repeats in CDS and lnTPM$_{max}$. **(b)** Regression model for the correlation between the density of AGG/CCT repeats in CDS and lnTPM$_{\mathbf{max}}$. **(c)** Regression model for the correlation between the density of ATC/ATG repeats in CDS and lnTPM$_{max}$. The asterisks indicate the optimal model ($p < 0.05$). The overfitting models and expMotif-region combinations with a sample size of less than 15 were excluded.

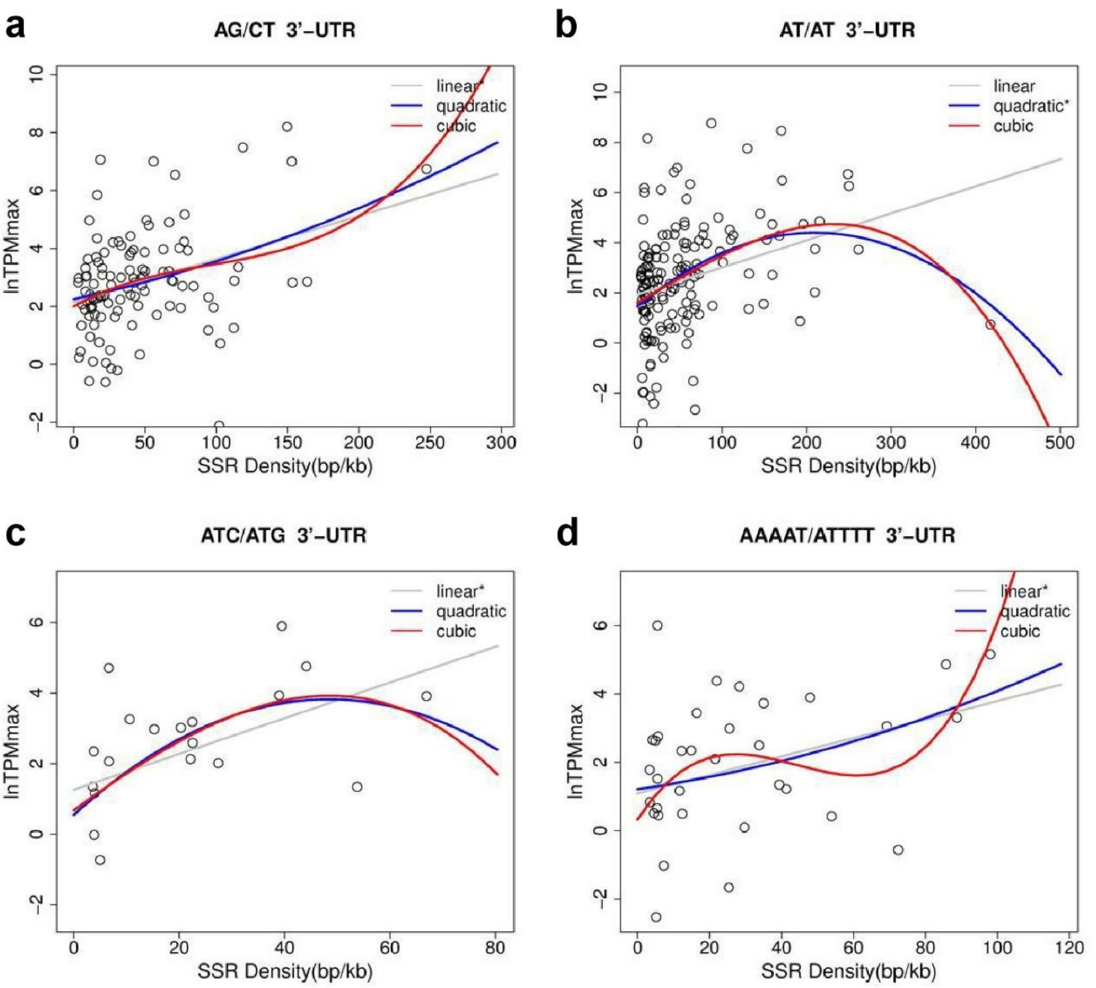

Figure S13. Regression models based on $\ln TPM_{max}$ and SSR density of fully standardized expMotifs within the 3'-UTR. **(a)** Regression model for the correlation between the density of AG/CT repeats in 3'-UTR and $\ln TPM_{max}$. **(b)** Regression model for the correlation between the density of AT/AT repeats in 3'-UTR and $\ln TPM_{\mathbf{max}}$. **(c)** Regression model for the correlation between the density of ATC/ATG repeats in 3'-UTR and $\ln TPM_{max}$. **(d)** Regression models for the correlation between the density of AAAAT/ATTTT repeats in 3'-UTR and $\ln TPM_{max}$. The asterisks indicate the optimal model ($p < 0.05$). The overfitting models and expMotif-region combinations with a sample size of less than 15 were excluded.

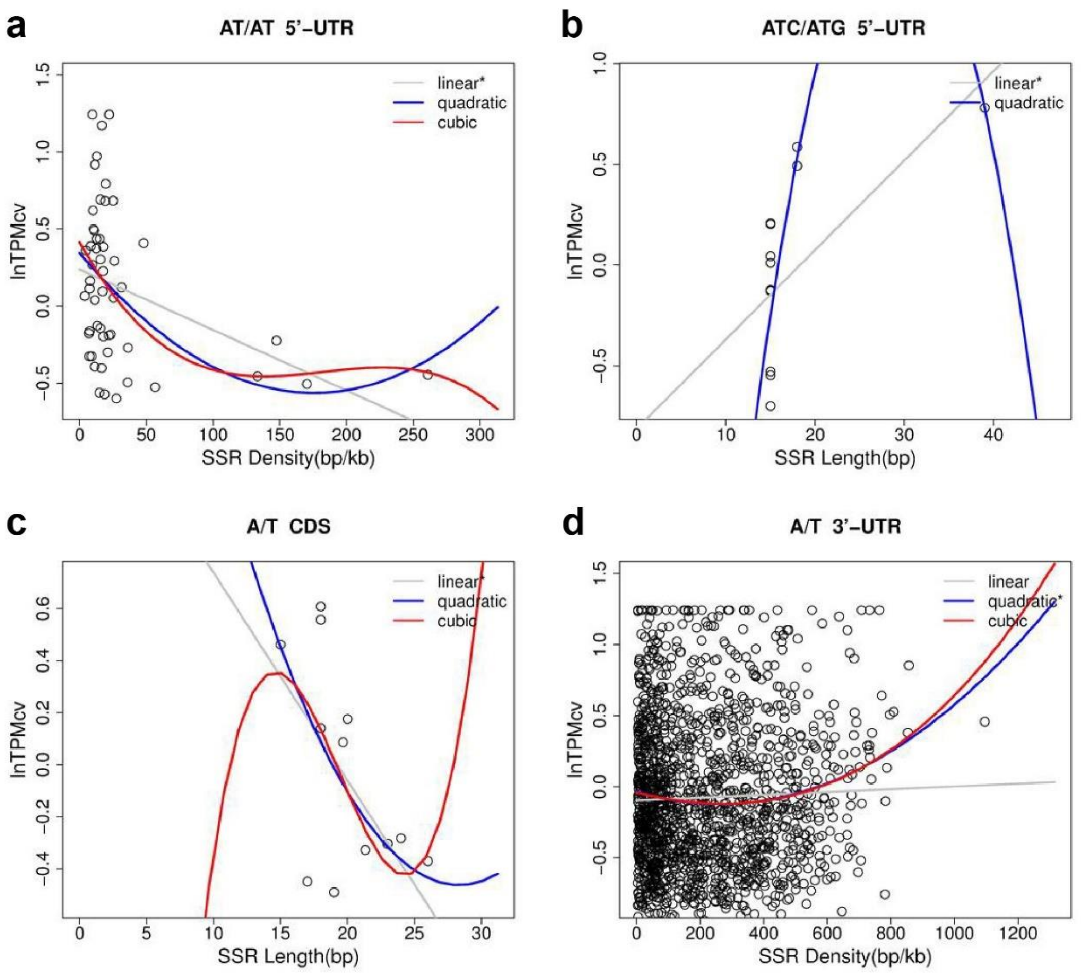

Figure S14. Regression models based on lnTPMCV and SSR density of fully standardized expMotifs within three transcribed regions. **(a)** Regression model for the correlation between the density of AT/AT repeats in 5'-UTR and lnTPMCV. **(b)** Regression model for the correlation between the length of ATC/ATG repeats in 5'-UTR and lnTPMCV. **(c)** Regression model for the correlation between the length of A/T repeats in CDS and lnTPMCV. **(d)** Regression models for the correlation between the density of A/T repeats in 3'-UTR and lnTPMCV. The asterisks indicate the optimal model ($p < 0.05$). The overfitting models and expMotif-region combinations with a sample size of less than 15 were excluded.

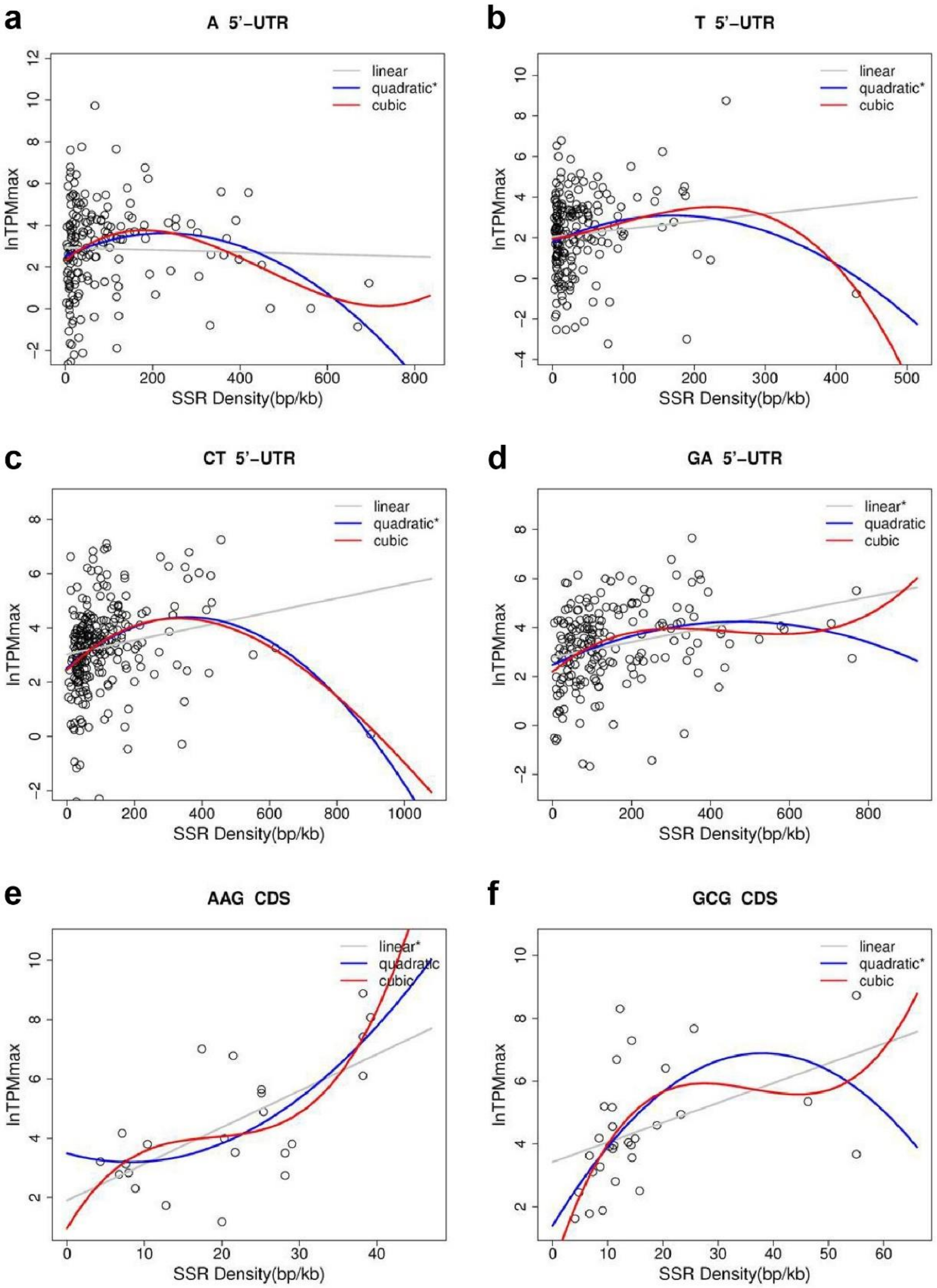

Figure S15. Regression models based on $lnTPM_{max}$ and SSR density of actual expMotifs within the 5'-UTR and CDS. **(a)** Regression model for the correlation between the density of T repeats in 5'-UTR and $lnTPM_{max}$. **(b)** Regression model for the correlation between the density of T repeats in 5'-UTR and $lnTPM_{\mathbf{max}}$. **(c)** Regression model for the correlation between the density of CT repeats in 5'-UTR

and $lnTPM_{max}$. **(d)** Regression models for the correlation between the density of GA repeats in 5'-UTR and $lnTPM_{max}$. **(e)** Regression model for the correlation between the density of AAG repeats in CDS and $lnTPM_{max}$. **(f)** Regression model for the correlation between the density of GCG repeats in CDS and $lnTPM_{max}$. The asterisks indicate the optimal model ($p < 0.05$). The overfitting models and expMotif-region combinations with a sample size of less than ten were excluded.

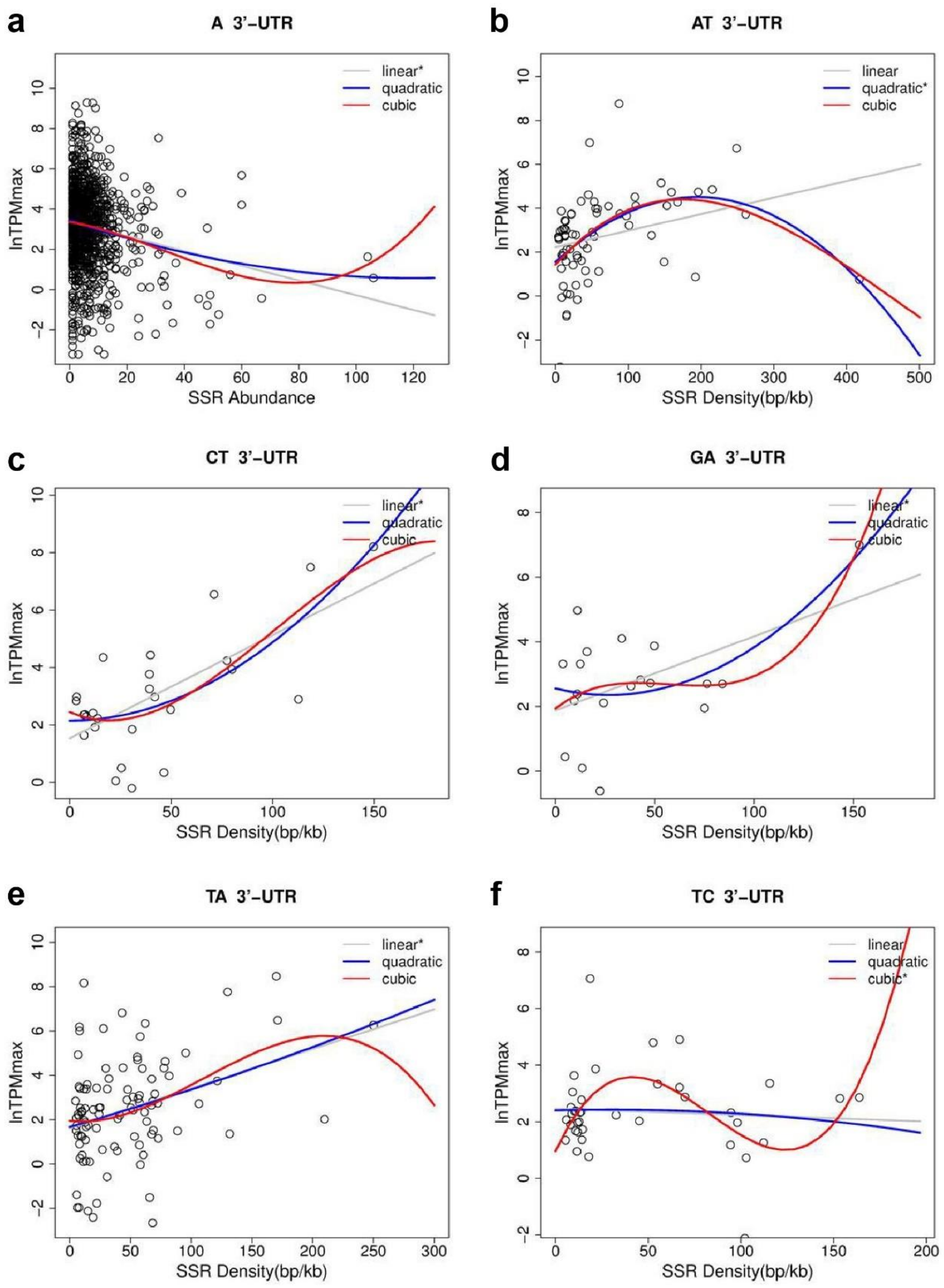

Figure S16. Regression models based on $lnTPM_{max}$ and SSR density of actual expMotifs within the 3'-UTR. **(a)** Regression model for the correlation between the abundance of A repeats in 3'-UTR and $lnTPM_{max}$. **(b)** Regression model for the

correlation between the density of AT repeats in 3’-UTR and $lnTPM_{max}$. **(c)** Regression model for the correlation between the density of CT repeats in 3’-UTR and $lnTPM_{max}$. **(d)** Regression models for the correlation between the density of GA repeats in 3’-UTR and $lnTPM_{max}$. **(e)** Regression model for the correlation between the density of TA repeats in 3’-UTR and $lnTPM_{max}$. **(f)** Regression model for the correlation between the density of TC repeats in 3’-UTR and $lnTPM_{max}$. The asterisks indicate the optimal model ($p < 0.05$). The overfitting models and expMotif-region combinations with a sample size of less than ten were excluded.

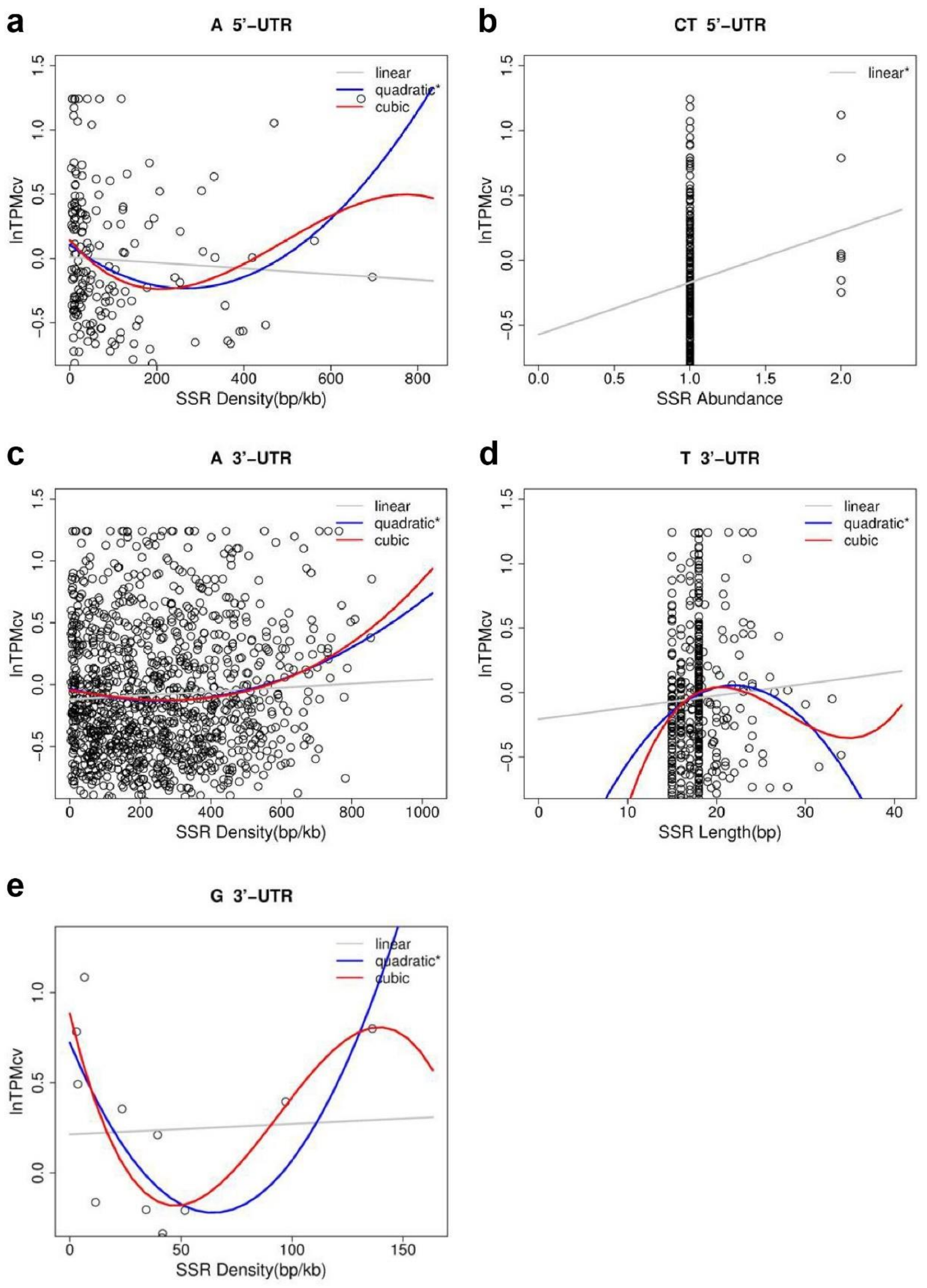

Figure S17. Regression models based on $\ln TPM_{CV}$ and SSR characteristics of actual expMotifs within transcribed regions. **(a)** Regression model for the correlation between the density of A repeats in 5'-UTR and $\ln TPM_{CV}$. **(b)** Regression model for the correlation between the abundance of CT repeats in 5'-UTR and $\ln TPM_{CV}$. **(c)**

Regression model for the correlation between the density of A repeats in 3’-UTR and lnTPM$_{CV}$. **(d)** Regression models for the correlation between the length of T repeats in 3’-UTR and lnTPM$_{CV}$. **(e)** Regression model for the correlation between the density of G repeats in 3’-UTR and lnTPM$_{CV}$. The asterisks indicate the optimal model ($p < 0.05$). The overfitting models and expMotif-region combinations with a sample size of less than ten were excluded.

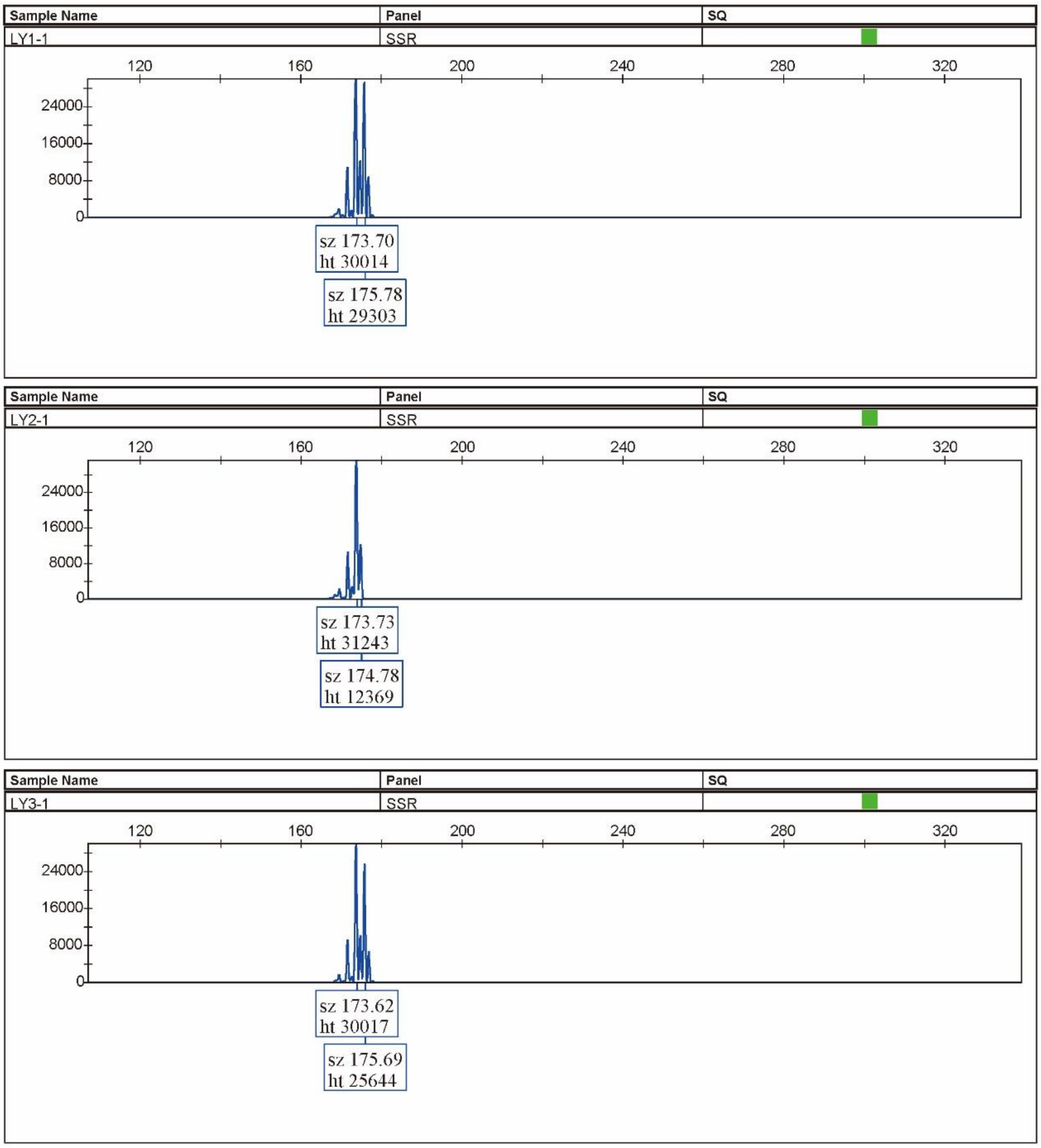

Figure S18. Profiles of expMotif-SSRs at TCP gene i1_HQ_lanhua_c24148/f3p1/1714 in three individuals by CE.

Additional file 1: Tables

Table S1. Statistics of PacBio sequencing of the P. malipoense transcriptome.

cDNA library	1-6 kb
**PacBio sequencing**	
Subreads base [Gb]	13.25
Subreads number	5,611,491
Average subreads length [bp]	2,362
N50 of subreads [bp]	2,712
**ROI dataset**	
CCS number	360,416
Number of 5'-primer reads	334,917 (92.93%)
Number of 3'-primer reads	336,419 (93.34%)
Number of poly-A reads	325,528 (90.32%)
Number of full-length reads	298,890 (82.93%)
Number of non-full-length reads	60,627 (16.82%)
Number of FLNC reads	283,475
Average FLNC read length [bp]	2,626
**ICE dataset**	
Consensus isoforms	126,513
Average consensus isoform length [bp]	2,792
N50 of consensus isoforms [bp]	2,954
**Hybrid correction dataset**	
Number of transcripts	126,513
Minimum length transcripts [bp]	201
Maximum length transcripts [bp]	14,425
Average length of transcripts [bp]	2,815
N50 of transcripts	2,967
**Unigenes**	
Number of transcripts	63,940
Minimum length transcripts [bp]	201
Maximum length transcripts [bp]	14,425
Average length of transcripts [bp]	3,044
N50 of transcripts [bp]	3,283

Table S2. Summary of the functional annotation of the reference transcriptome of P. malipoense.

**Databases**	**Number of Unigenes**	**Percentage [%]**
Nr	54,009	84.47
Swiss-Prot	44,373	69.4
eggNOG	53,468	83.62
KOG	39,146	61.22
GO	43,773	68.46
KEGG	28,525	44.61
Pfam	47,370	74.09
Annotated in all databases	24,457	38.25
Annotated in at least one database	55,116	86.2
Total	63,940	100

Table S3. Kruskal–Wallis test results of TPM_{max} among transcribed regions with different motif sizes of SSRs.

**Motif size**	χ^2	**df**	***p* value**	**Median of TPM_{max}**			
				**5'-UTR**	**CDS**	**3'-UTR**	**None**
Mono-	26.596	3	7.16E-06	16.100^{b}	9.260^{b}	22.080^{a}	21.660^{a}
Di-	19.748	2	5.15E-05	25.050^{a}	8.030^{*}	14.880^{c}	21.610^{b}
Tri-	22.805	3	4.43E-05	21.890^{b}	27.740^{a}	18.180^{b}	21.300^{b}
Tetra-	4.436	2	0.1088	29.02	6.585^{*}	15.85	21.64
Penta-	39.235	3	1.55E-08	14.720^{bc}	21.760^{ab}	9.075^{c}	21.795^{a}
Hexa-	17.852	3	4.72E-04	16.950^{cb}	30.315^{a}	9.695^{c}	21.645^{ab}

Note: Different superscript letters represent significant differences, and the same superscript letters represent no significant difference (one-side test, BH adjusted *p* value < 0.05). The asterisks indicate this group was excluded from the test due to the sample size being less than ten within CDS or less than 20 within UTRs.

Table S4. Kruskal–Wallis test results of TPM_{CV} among transcribed regions with different motif sizes of SSRs.

Motif size	χ^2	df	*p* value	Median of TPM_{CV}			
				5'-UTR	CDS	3'-UTR	None
Mono-	140.24	3	< 2.2e-16	0.897^a	1.151^a	0.860^b	0.763^c
Di-	6.031	2	0.049	0.774^{ab}	0.974^*	0.840^a	0.773^b
Tri-	1.76	3	0.6237	0.8	0.773	0.781	0.774
Tetra-	4.977	2	0.0831	0.696	0.832^*	0.831	0.774
Penta-	12.258	3	0.0065	0.907^a	0.787^{ab}	0.813^{ab}	0.773^b
Hexa-	3.755	3	0.2892	0.802	0.749	0.88	0.773

Note: Different superscript letters represent significant differences, and the same superscript letters represent no significant difference (one-side test, BH adjusted p value < 0.05). The asterisks indicate indicates this group was excluded from the test due to the sample size being less than ten within CDS or less than 20 within UTRs.

Table S5. Fully standardized motifs distributed in different transcribed regions had significantly different TPM_{max} values.

Repeat types	χ^2	df	*p* value	Median of TPM_{max}			
				5'-UTR	CDS	3'-UTR	None
A/T	17.222	3	6.36E-04	16.255^b	11.960^b	22.930^a	21.620^{ab}
C/G	13.251	3	4.12E-03	22.590^{ab}	9.220^{ab}	7.530^b	21.650^a
AT/AT	43.785	2	3.11E-10	5.460^c	1.980^*	12.640^b	21.840^a
AG/CT	22.114	2	1.58E-05	27.180^a	51.575^*	15.710^b	21.400^b
AAG/CTT	10.128	2	6.32E-03	28.045^{ab}	31.580^a	18.330^*	21.530^b
CCG/CGG	16.954	2	2.08E-04	22.625^{ab}	32.630^a	37.890^*	21.480^b
AAAAT/ATTTT	-	-	6.70E-04	22.260^*	-	8.15	21.655

Note: Different superscript letters represent significant differences, and the same superscript letters represent no significant difference (one-side test, BH adjusted p value < 0.05). The asterisks indicate indicates this group was excluded from the test due to the sample size being less than ten within CDS or less than 20 within UTRs.

Table S6. Fully standardized motifs distributed in different transcribed regions had significantly different TPM_{CV} values.

Repeat types	χ^2	df	*p* value	Median of TPM_{CV}			
				5'-UTR	CDS	3'-UTR	None
A/T	92.786	3	< 2.2e-16	0.892^{a}	0.754^{ab}	0.855^{a}	0.765^{b}
AT/AT	24.239	2	5.45E-06	1.101^{a}	1.025^{*}	0.903^{b}	0.772^{c}
C/G	20.479	3	1.35E-04	0.841^{ab}	1.217^{a}	1.009^{a}	0.773^{b}
CCG/CGG	9.1026	2	1.06E-02	0.660^{b}	0.764^{ab}	0.876^{*}	0.774^{a}
ATC/ATG	6.8218	2	3.30E-02	1.011^{*}	0.665^{b}	0.732^{ab}	0.774^{a}

Note: Different superscript letters represent significant differences, and the same superscript letters represent no significant difference (one-side test, BH adjusted p value < 0.05). The asterisks indicate indicates this group was excluded from the test due to the sample size being less than ten within CDS or less than 20 within UTRs.

Table S7. The optimal models of fully standardized motif characteristics and $lnTPM_{max}$.

Motif	Region	The best model	R^2	*p* value	BIC	Maximum VIF
A/T	5'-UTR	y=1.097e-02den-2.201e-05den^2+2.157e+00	0.0517	1.98E-04	1381.65	-
	3'-UTR	y=-0.058782abd+0.001877den+2.980853	0.0264	1.31E-09	6454.81	1.9469
C/G	5'-UTR	y=0.008021den+2.078891	0.1481	1.55E-02	175.8	-
AG/CT	5'-UTR	y=1.227e-02den-2.640e-05den^2+1.523e-08den^3+2.358e+00	0.0901	3.16E-15	2838.26	-
	3'-UTR	y=0.01486den+2.14875	0.1206	2.01E-04	435.47	-
AT/AT	3'-UTR	y=2.802e-02den-6.679e-05den^2+1.464e+00	0.1461	2.78E-06	721.72	-
AAG/CTT	5'-UTR	y=0.0263814den-0.0000584den^2+1.9179038	0.3354	2.99E-05	187.72	-
	CDS	y=0.05531den+2.86448	0.114	3.26E-03	297.64	-
AGG/CCT	5'-UTR	y=0.0706666den-0.0002921den^2	0.7658	4.50E-12	153.4	-
	CDS	y=0.50051den-0.01502den^2	0.8411	< 2.2e-16	520.61	
ATC/ATG	CDS	y=0.171247den-0.003637den^2+1.946638	0.1075	3.59E-03	377.31	-
	3'-UTR	y=0.05063den+1.26034	0.2132	4.04E-02	88.33	-
CCG/CGG	CDS	y=0.08212den-0.08594len+3.93348	0.1514	5.76E-07	683.78	1.0923
AAAAT/ATTTT	3'-UTR	y=0.02703den+1.08876	0.1196	4.19E-02	157.17	-

Table S8. The optimal models of fully standardized motif characteristics and $lnTPM_{CV}$.

Motif	Region	The best model	R^2	*p* value	BIC
A/T	CDS	y=-0.07987len+1.53716	0.3296	4.02E-02	16.31
	3’-UTR	y=-6.747e-04den+1.287e-06den^2-3.510e-02	0.0112	1.81E-04	2349.75
AT/AT	5’-UTR	y=-0.003927den+0.237735	0.1085	1.41E-02	93.36
ATC/ATG	5'-UTR	y=0.04438len-0.81243	0.3314	3.95E-02	20.8

Table S9. Summary of the results of statistical tests and regression analyses of 29 candidate expMotifs.

Fully standardized motif	Actual motif	Region	Number of SSR-containing Sequences	TPM_{max}		TPM_{CV}	
				Statistical test	Regression analysis	Statistical test	Regression analysis
A/T	A	5'-UTR	194		*	*	*
		3'-UTR	1182	*	*	*	*
	T	5'-UTR	216	*	*	*	
		CDS	11		*		
		3'-UTR	473	*		*	*
C/G	C	5'-UTR	24		*		*
		3'-UTR	31				
	G	5'-UTR	15		*		
		3'-UTR	13	*		*	*
AG/CT	AG	5'-UTR	159		*		
		3'-UTR	35		*		
	GA	5'-UTR	195	*	*		
		3'-UTR	19		*		
	CT	5'-UTR	256	*	*		*
		3'-UTR	26	*	*	*	
	TC	5'-UTR	167		*		*
		3'-UTR	40	*	*		
AT/AT	AT	5'-UTR	29	*			*
		3'-UTR	70		*		
	TA	5'-UTR	31	*		*	
		3'-UTR	100	*	*	*	

AAG/CTT	AAG	CDS	23	*	*		
	AGA	5'-UTR	13		*		
		CDS	11				*
	GAA	5'-UTR	12				
		CDS	28				
	TTC	5'-UTR	11		*		
AGG/CCT	AGG	CDS	26		*	*	
	GAG	CDS	25				
	GGA	CDS	11				*
	CCT	CDS	15	*			
	CTC	5'-UTR	11		*		
		CDS	34		*		
	TCC	CDS	34		*		
ATC/ATG	ATG	CDS	45				
	GAT	CDS	28				
	TGA	CDS	27		*		
CCG/CGG	CCG	5'-UTR	18				
		CDS	51				
	CGC	5'-UTR	12				*
		CDS	21				
	GCC	5'-UTR	12		*		
		CDS	33		*		
	CGG	CDS	28	*			
	GCG	CDS	30	*	*		
	GGC	CDS	27		*		

Note: The statistical test was the Kruskal–Wallis test (and Dunn's post hoc tests) or Mann–Whitney test. The * indicate statistical significance of corresponding to p value < 0.05. The TPM_{max}- and TPM_{CV}-associated expMotifs were highlighted in yellow and blue respectively.

Table S10. Actual motifs distributed in different transcribed regions had significantly different TPM_{max} values.

Repeat types	χ^2	df	*p* value	Median of TPM_{max}			
				5'-UTR	CDS	3'-UTR	None
A	11.718	2	2.85E-03	20.870[ab]	9.260*	25.630[a]	21.300[b]
T	47.594	2	4.63E-11	10.740[c]	11.185*	15.770[b]	21.970[a]
G	9.183	2	1.01E-02	8.040[ab]	23.460*	6.545[b]	21.640[a]
AT	17.665	2	1.46E-04	5.610[b]	7.470*	16.095[a]	21.720[a]
TA	27.227	2	1.22E-06	5.460[b]	1.980*	11.110[b]	21.750[a]
GA	-	-	1.72E-02	30.45	3.190*	14.900*	21.55
CT	9.255	2	9.78E-03	28.590[a]	51.460*	14.735 [ab]	21.490[b]
TC	9.446	2	8.89E-03	23.540[a]	80.075*	10.000[b]	21.650[a]
AAG	-	-	2.22E-03	26.180*	45.12	58.315*	21.57
CCT	-	-	9.37E-04	15.090*	62.7	-	21.595
CGG	-	-	4.50E-03	42.310*	41.745	103.000*	21.555
GCG	-	-	1.24E-04	13.190*	54.98	-	21.58
GAT	-	-	4.55E-02	6.870*	32.22	-	21.6

Note: Different superscript letters represent significant differences, and the same superscript letters represent no significant difference (one-side test, BH adjusted p value < 0.05). The asterisks indicates this group was excluded from the test due to the sample size being less than ten.

Table S11. Actual motifs distributed in different transcribed regions had significantly different TPM_{CV} values.

Repeat types	χ^2	df	*p* value	Median of TPM_{CV}			
				5'-UTR	CDS	3'-UTR	None
A	56.064	2	6.70E-13	0.895[a]	0.721[*]	0.844[a]	0.769[b]
T	68.988	2	1.05E-15	0.940[a]	0.922[*]	0.904[b]	0.769[c]
G	8.714	2	1.28E-02	0.758[ab]	1.434[*]	1.330[a]	0.774[b]
TA	19.536	2	5.73E-05	1.101[a]	1.048[*]	0.927[a]	0.773[b]
CT	6.672	2	3.56E-02	0.795[ab]	0.520[*]	1.099[a]	0.774[b]
AGG	-	-	1.11E-02	1.032[*]	1.08	-	0.775[b]

Note: Different superscript letters represent significant differences, and the same superscript letters represent no significant difference (one-side test, BH adjusted p value < 0.05). The asterisks indicates this group was excluded from the test due to the sample size being less than ten.

Table S12. The optimal models of actual motif characteristics and $lnTPM_{max}$.

Motif	Region	The best model	R^2	*p* value	BIC	Maximum VIF
A	5'-UTR	y=0.009632den-0.000021den^2+2.516505	0.057	5.22E-03	806.45	-
	3'-UTR	y=-0.03661abd+3.38152	0.023	1.87E-07	4923.24	-
T	5'-UTR	y=1.529e-02den-4.505e-05den^2+1.797e+00	0.0374	2.52E-02	842.8	-
	CDS	y=-0.8957abd+0.0838den+2.6777	0.9279	1.40E-03	19.87	1
C	5'-UTR	y=0.0509584den-0.0001508den^2+0.6930629	0.3009	2.33E-02	115.22	-
G	5'-UTR	y=0.007718den+1.778244	0.4348	7.49E-03	55.04	-
AG	5'-UTR	y=6.428e-03den-6.080e-06den^2+2.629e+00	0.0825	1.21E-03	619.46	-
	3'-UTR	y=0.01897den+2.33830	0.2238	4.10E-03	134.89	-
GA	5'-UTR	y=0.003077den+2.789853	0.0681	2.30E-04	757.8	-
	3'-UTR	y=0.02288den+1.88080	0.2494	2.95E-02	76.74	-
CT	5'-UTR	y=1.058e-02den-1.486e-05den^2+2.523e+00	0.1081	5.22E-07	964.85	-
	3'-UTR	y=0.03594den+1.53030	0.4635	1.29E-04	103.91	-
TC	5'-UTR	y=1.360e-02den-2.599e-05den^2+2.232e+00	0.0967	2.66E-04	631.49	-
	3'-UTR	y=1.425e-01den-2.290e-03den^2+9.221e-06den^3+9.566e-01	0.2079	4.52E-02	143.86	-
AT	3'-UTR	y=3.023e-02den-7.738e-05den^2+1.562e+00	0.2481	7.11E-05	285.58	-
TA	3'-UTR	y=0.01783den+1.62122	0.1132	6.20E-04	454.9	-
AAG	CDS	y=0.1232den+1.9000	0.4441	5.17E-04	94.06	-
AGA	5'-UTR	y=0.01631den+2.66767	0.4714	9.55E-03	42.79	-
TTC	5'-UTR	y=0.02581den+1.98207	0.4552	2.28E-02	34.11	-
AGG	5'-UTR	y=-0.1507den+9.3533	0.7098	3.53E-02	20.41	-
	CDS	y=0.2436den+0.6567	0.1906	2.57E-02	113.77	-
CTC	5'-UTR	y=-0.3356len+9.5405	0.5061	1.41E-02	44.04	-

	CDS	y=0.1588den+1.6845	0.2223	4.88E-03	131.97	-
TCC	CDS	y=0.36969den-0.01302den^2+1.31778	0.2425	1.35E-02	129.61	-
GCC	5'-UTR	y=0.04927den-0.00018den^2+0.97402	0.5369	3.13E-02	36.26	-
	CDS	y=0.1054den+1.7735	0.2171	6.27E-03	120.33	-
GCG	CDS	y=0.28947den-0.00381den^2+1.39498	0.3238	5.08E-03	123.57	-
GGC	CDS	y=0.1831den-0.1084len+3.3768	0.5572	5.69E-05	76.12	1.4212
TGA	CDS	y=0.1377den+1.9038	0.4291	2.09E-04	85.92	-

Table S13. The optimal models of actual motif characteristics and lnTPM$_{CV}$.

Motif	Region	The best model	R^2	*p* value	BIC	Maximum VIF
A	5'-UTR	y=-2.556e-03den+4.822e-06den^2+1.063e-01	0.0507	9.50E-03	300.02	-
	3'-UTR	y=-7.569e-04den+1.467e-06den^2-3.306e-02	0.0154	1.19E-04	1805.22	-
T	3'-UTR	y=0.186283len-0.004251len^2-1.984654	0.0196	1.14E-02	697.29	-
C	5'-UTR	y=-2.137e-02den+1.320e-04den^2-2.245e-07den^3+0.585	0.5135	2.05E-03	30.25	-
G	3'-UTR	y=-0.0293138den+0.0002278den^2+0.7217928	0.5853	1.91E-02	15.43	-
AT	5'-UTR	y=-0.003397den+0.171732	0.1432	4.30E-02	51.74	-
CT	5'-UTR	y=0.4008abd-0.5719	0.0209	2.06E-02	331.1	-
TC	5'-UTR	y=0.0007961den-0.2210027	0.0362	1.43E-02	237.82	-
AGA	CDS	y=-0.1139den+1.2754	0.5975	5.27E-03	13.36	-
GGA	CDS	y=0.08107den-1.37545	0.4119	3.33E-02	11.3	-
CGC	5'-UTR	y=0.1182len-2.4264	0.5723	7.05E-03	7.28	-

Table S14. Groping criteria for unigenes based on TPM_{max} and TPM_{CV} values.

level	TPM_{max}		TPM_{CV}	
	cut-off	sequence counts	cut-off	sequence counts
1	TPM_{max} >= 1000	394	TPM_{CV} >= 3.4641	1,229
2	1000 > TPM_{max} >= 100	3,264	3.4641 > TPM_{CV} >= 1.5	8,933
3	100 > TPM_{max} >= 10	16,520	1.5 > TPM_{CV} >= 1	15,865
4	10 > TPM_{max} >= 1	24,215	1 > TPM_{CV} >= 0.5	30,841
5	TPM_{max} < 1	15,429	TPM_{CV} < 0.5	2,951

Table S15. Primers used for qRTPCR.

Gene	Primers(5'-3')	Product length (bp)
i1_HQ_lanhua_c24148/f3p1/1714	F: CCTCCCGTCCTTCATCGTTC	100
	R: CATCGGAAGACGACTCCTGG	
i1_LQ_lanhua_c47139/f1p12/1518	F: CATGACGACTGGTCTGCACT	142
	R: AGGTCGGTAAGAGTTCAGCG	
i1_LQ_lanhua_c20715/f1p0/1521	F: GCAGAGAGCTGCAAATCGTG	142
	R: GTGTGACGGTCCTTGTTGGA	
i1_LQ_lanhua_c8646/f1p9/1748	F: CTGGCCTCAACCCCAAACTC	199
	R: TGCCCACTTAGCAATGAACCA	
ACTIN	F: GAGAAGCTGGCGTATGTTGC	176
	R: GCAGCTTCCATGCCAATCAG	

Additional file 1: Datas

Data 1. Illumina sequencing quality of seven RNA-seq transcriptomes sequenced in this study (see separate files)**.**

Data 2. TPM matrix and groping information of unigenes based on TPM_{max} and TPM_{CV} values (see separate files)**.**

Data 3. Results of qRTPCR (see separate files)**.**
